# Biomolecular simulation based machine learning models accurately predict sites of tolerability to the unnatural amino acid acridonylalanine

**DOI:** 10.1038/s41598-021-97965-2

**Published:** 2021-09-15

**Authors:** Sam Giannakoulias, Sumant R. Shringari, John J. Ferrie, E. James Petersson

**Affiliations:** 1 Department of Chemistry, University of Pennsylvania, 231 S. 34th St, Philadelphia, PA 19104 USA; 2 Department of Molecular & Cell Biology, University of California, Berkeley, 475B Li Ka Shing Center, Berkeley, CA 94720 USA

**Keywords:** Computational science, Computational biophysics

## Abstract

The incorporation of unnatural amino acids (Uaas) has provided an avenue for novel chemistries to be explored in biological systems. However, the successful application of Uaas is often hampered by site-specific impacts on protein yield and solubility. Although previous efforts to identify features which accurately capture these site-specific effects have been unsuccessful, we have developed a set of novel Rosetta Custom Score Functions and alternative Empirical Score Functions that accurately predict the effects of acridon-2-yl-alanine (Acd) incorporation on protein yield and solubility. Acd-containing mutants were simulated in PyRosetta, and machine learning (ML) was performed using either the decomposed values of the Rosetta energy function, or changes in residue contacts and bioinformatics. Using these feature sets, which represent Rosetta score function specific and bioinformatics-derived terms, ML models were trained to predict highly abstract experimental parameters such as mutant protein yield and solubility and displayed robust performance on well-balanced holdouts. Model feature importance analyses demonstrated that terms corresponding to hydrophobic interactions, desolvation, and amino acid angle preferences played a pivotal role in predicting tolerance of mutation to Acd. Overall, this work provides evidence that the application of ML to features extracted from simulated structural models allow for the accurate prediction of diverse and abstract biological phenomena, beyond the predictivity of traditional modeling and simulation approaches.

## Introduction

Expansion of the genetic code by incorporation of unnatural amino acids (Uaas) has helped to facilitate the study of biochemical phenomena which would otherwise be elusive^1–4^. Although Uaa incorporation is often used for the direct expression and purification of proteins with specific post-translational modifications, where the site would be dictated by biological relevance, Uaas are also used for photo-crosslinking, spectroscopic labeling, and biorthogonal conjugation, where there are many options for the location of the Uaa^5–9^. Various studies, including our own, have demonstrated that the specific incorporation site of a Uaa has dramatic impacts on both the solubility and yield of the resultant mutant protein^10–12^. However, identification of positions which will tolerate the newly incorporated Uaa is nontrivial.

A predictive method which can rapidly and accurately identify sites for Uaa incorporation that maximize mutant protein solubility and yield could dramatically increase the use of Uaas in both academia and industry. Several computational efforts have focused on accurately predicting structural aspects of Uaa mutant proteins, such as amino acid rotameric or backbone orientations^13–16^. Others have concentrated on predicting interaction phenomena such as protein–protein binding affinities^17,18^ or energies of hydration^19^, but none have successfully predicted more complex phenomena such as Uaa protein yield and soluble fraction. We believe the lack of attention dedicated to these predictions stems from both the absence of a robust dataset that contains uniform information regarding a protein’s native structure, solubility, and yield and a lack of evidence supporting the predictability of such phenomena^10^. Ultimately, in *lieu* of an effective predictor, Uaa incorporation has often been restricted to sites where native residues possess similar chemical characteristics to the Uaa of interest^20^ or to mutationally tolerant sites identified prior to Uaa incorporation^21^. Alternative approaches employ empirical screening of sites, often through the use of a green fluorescent protein (GFP) fusion reporter system to assess Uaa incorporation efficiency^22,23^. The former approaches are very limiting in the number of positions for Uaa incorporation, and the latter approach can require effort comparable to or greater than the effort needed for the eventual experiment with the Uaa-labeled protein. Thus, there is a great need for a facile approach which can identify sites in proteins that will tolerate mutation to Uaas. A demonstration of a simple predictive method may also encourage community wide data collection and result in a sufficiently large and varied dataset which would serve as a major step for improve Uaas predictability.

Previously, we collected the largest uniform dataset that captures the soluble yield, total yield, and soluble fraction for a singular unnatural amino acid (acridon-2-ylalanine, Acd) in a pair of protein targets^10^. During that investigation, we attempted to develop a simple heuristic descriptor which could predict the effects of Uaa incorporation on these measurables, but were unsuccessful. Acd, a blue wavelength fluorophore, was selected for this study because of its ability to be used as an intrinsically fluorescent Uaa, its ability to be assayed quickly and cleanly using gel-electrophoresis, and its many uses in in vitro assays such as fluorescence polarization and FRET experiments as well as recent applications in live cell imaging^24–29^. Acd has been shown to fulfill these functions at a variety of positions in proteins and exemplifies the problem of choosing an insertion site that is tolerated by the target protein. Our previous effort focused on investigating the ability of structure-independent bioinformatics-based features (BLOSUM62 matrix, evolutionary conservation, measures of local hydrophobicity, etc.) to act as heuristic predictors of the soluble fraction for various Acd-containing mutants of the bacterial proteins LexA and RecA^10^. However, we demonstrated that none of the tested structure-independent bioinformatics features individually acted as reliable predictors of tolerability, as none displayed a Pearson or Point-Biserial correlation coefficient (R, calculated with SciPy) above 0.25 with the Acd mutant soluble fraction data for LexA or RecA independently, or for the combined set. Interestingly, the most useful features identified were categorical variables corresponding to the domain and secondary structure in which Acd was incorporated. Although these heuristics seemed to be relatively descriptive for LexA, which is composed of two isolated domains with different secondary structures (an α-helical N-terminal domain and a β-sheet C-terminal domain connected by a flexible linker), the trend did not hold for RecA, which comprises multiple mixed α/β domains. Lastly, we investigated the utility of using the scores of structures resulting from Backrub simulations of the Acd mutant proteins in Rosetta, which were again unable to act as effective predictors of tolerability to Acd^10^. Overall, this suggested that additional attention was required to identify predictive features for this dataset that could support generalization, prior to developing higher throughput methods for expanding the dataset.

**Figure 1:**
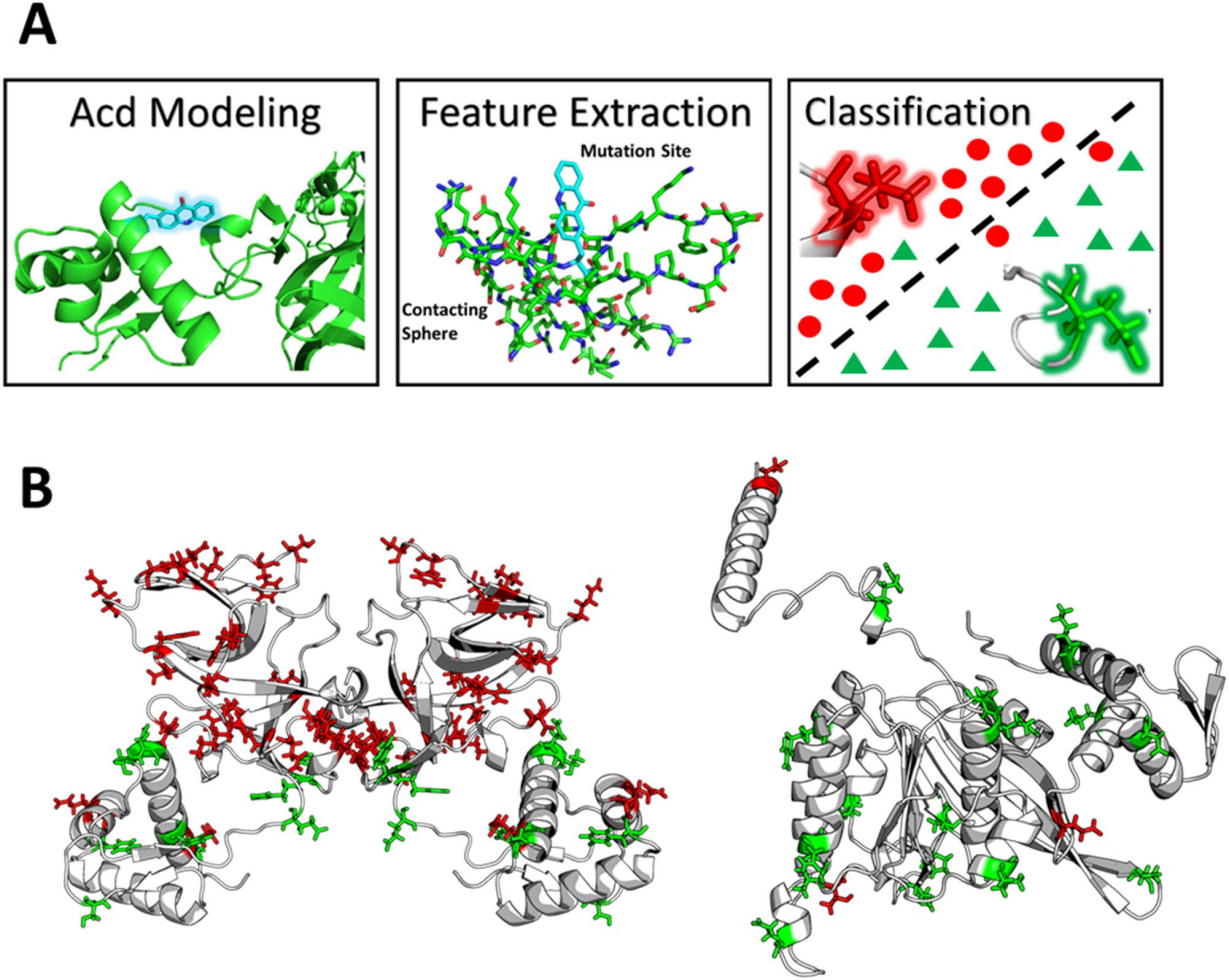
Schematic of the computational workflow for developing a Rosetta Custom Score Function or Empirical Score Function (**A**), spatial distribution and effect on soluble fraction of Acd mutants (**B**). LexA homo-dimer (left), RecA monomer (right). Note, red corresponds to soluble fraction percentage equal to or below 39%, and green above 39%.

Herein, we focus on establishing an accurate method for predicting Acd mutant protein soluble fraction (soluble yield divided by total yield). This metric helps to report on whether mutation of a residue to Acd will be tolerated and represents a class of experiments that has evaded predictive methods in the past. Previously, we demonstrated that the predictivity of Rosetta methods can be dramatically improved through the use of RCSFs^30,31^. RCSFs, or Rosetta Custom Score Functions, rely on generation of structural models in PyRosetta, which are subsequently scored with the Rosetta full atom score function (beta_nov_16)^32^, a linear combination of energetic score terms (Lennard–Jones potential, electrostatics, implicit solvation etc.) that serves an analogous role to forcefields in molecular dynamics (MD) simulations. Isolated score terms are then subsequently re-combined through machine learning (ML) to generate an RCSF (Fig. 1A). Given the adaptability of RCSFs, we sought to investigate their utility in this problem that has previously proved difficult. First, we focused on determining if the constitutive energies of the Rosetta score function are more correlative than the structure independent bioinformatics terms we previously tested. We also wished to test the descriptive capacity of combining these terms through multiple linear regression (MLR). Subsequently, we sought to determine if the correlative nature of these features was unique to the energetic terms in Rosetta, by investigating a set of Empirical Score Terms (ESTs) which are based on contacts and structure independent bioinformatics. After identifying both Rosetta and EST features that demonstrated significantly improved correlation, we then used ML to train RCSFs and Empirical Score Functions (ESFs) and compare their ability to predict Acd mutant protein solubility and yield. Lastly, we performed feature importance analysis of the most predictive models from both the RCSF and ESF methods to see which features imbue predictivity in order to better understand our system. Overall, this effort demonstrates that such ML approaches are able to predict complex phenomena related to Uaa incorporation.

## Methods

In order to simulate the Acd mutant LexA and RecA proteins, we first preprocessed and energy minimized the LexA and RecA protein structures (from PDB IDs 1JHH^33^ and 3CMW^34^, respectively) as detailed in the Starting Structures section of the Supplementary Information^35^. The energy minimized parent structures of LexA and RecA were then mutated to incorporate Acd at previously experimentally-tested positions using PyRosetta. The Rosetta amino acid params and side chain rotamer library files used to make Acd mutant proteins were those used in our previous work^10^. Following mutation to Acd, the structures were subjected to five independent cartesian FastRelax simulations (protocol to achieve low-energy protein backbone and side-chain conformations similar to the starting conformation through iterative stages of packing and minimization, with increasing repulsive weight in the scoring function over the course of the simulation), where only residues with a an alpha carbon to alpha carbon (C_⍺_-C_⍺_) distance within 8 Å of Acd were allowed to be refined^31^. These “local relaxes” allow for the surrounding residues of the mutation site to accommodate the newly incorporated Acd residue and have previously been shown to be a good sampling scheme for protein design^36^. Every position in LexA and RecA which was mutated to Acd was also locally relaxed about the wildtype (WT) residue in order to generate the control scores.

The locally relaxed structures were scored with the beta_nov16 score function, selected for its previously demonstrated efficacy, and the energy terms from the score function were averaged over the five simulations on a per residue basis^31,32,36^. Score differences (deltas) were computed for the total score function and for each term between the average weighted scores for the Acd mutant and the corresponding WT values. Features for RCSF training were then passed as the score deltas at the mutation site as well as the average of the score deltas of the surrounding locally relaxed residues (Fig. 1A).

In addition to computing Rosetta energy features from our structural models, we sought to construct a second, more generally applicable, feature set for ML comprised of contact-based terms^10^. Relevant contacts (pairwise atom distances < 4 Å) were computed from our structural models on an intra- and inter-residue basis using the biopython library^37^. The score deltas corresponding to the change in contacts upon mutation were used as features for training ESFs. The contact-based features were supplemented with structure independent bioinformatics features which provide information of evolutionary conservation and various measures of physiochemical properties (BLOSUM matrices, conservation terms, measure of hydrophobicity etc.). Supplementary Table 3 describes each of the contact-based terms as well as the structure independent bioinformatics features in our ESF feature matrix.

The experimental dataset was prepared for ML by first assigning a response class to each sample based on the distribution of the dependent variable. In Fig. 1B, we present the spatial distribution of the Acd mutants and the effect they have on LexA and RecA solubility. Response class assignment was performed by identifying cutoffs which naturally balance the distribution of actives and inactives of the set. For soluble yield, total yield and soluble fraction the response classes were balanced at 520 nM, 1600 nM, and 39%, respectively. Next, to ensure that our ML models were not overfit or the product of specifically engineered hyperparameters, we created a large, well-balanced holdout dataset for validating our models. The complete dataset spans 51 datapoints where 32 points are mutations in LexA and the remaining 19 are from RecA^10^. The holdout dataset (not seen by the ML algorithms during hyperparameter tuning) was constructed to represent 20% of the total dataset, comprising equal amounts of data from both proteins with a representative distribution of soluble fraction values. Members of the holdout datasets can be found in Supplementary Table 1 and on our GitHub (https://github.com/ejp-lab/EJPLab_Computational_Projects/tree/master/RML_ACD/Dataset).

Given the large number of computed features to be used in ML, dimensionality was reduced by selecting important features with univariate statistical analysis with the SelectKBest module in scikit-learn^38^. The following ML algorithms were employed using the respective default parameters within scikit-learn to coarsely assess the effect of prediction accuracy as a function of the number of features: Logistic Regression (LOG), Kernel Ridge Regression (KRR), Linear Discriminant Analysis (LDA), Quadratic Discriminant Analysis (QDA), Support Vector Machines (SVC), K Nearest Neighbors (KNN), Bernoulli Naïve Bayes (BNB), Gaussian Naïve Bayes (GNB), and Gaussian Process Classification (GPC)^38^. The optimal number of features were selected as the combination of features which showed the highest accuracy following stratified fivefold cross-validation, or CV5. The holdout datasets were validated by the aforementioned algorithms that were tuned using stratified CV5 in an exhaustive grid search. Finally, tuning parameters can be found in Supplementary Tables 10 and 11.

The metrics we have used to validate the performance of our models are accuracy, precision, recall, and the F1 score. Accuracy scores represent the ratio of correctly predicted observations (true positives and true negatives) to the total number of observations. Precision is defined as the ratio of the number of correctly predicted positives to the total number of positive observations predicted. Recall is used to assess how many of the positive observations were identified and is given by the ratio of correctly predicted positives to total positives. Finally, the F1 score is the weighted average of precision and recall.

## Results

In our previous study, analysis of the backrub simulated structures demonstrated that neither structural deviations nor total energetic differences were correlative with any of the experimental parameters of interest^10^. To confirm that this was not an artifact of the sampling approach previously utilized, the experimental data from our previous study were simulated in PyRosetta as described in “Methods” section^10^ In this study, alpha carbon root mean squared deviation (C⍺RMSD) analysis was performed for locally relaxed structures and demonstrated that across the sets of independent simulations, each Uaa position converged to a singular structure in both the Acd mutant and WT simulations. The largest observed C⍺RMSD within a simulation set was 1.37 Å. Larger deviations of up to 4.07 Å were observed between the lowest energy member of a set of Acd mutant and WT simulations for a given position. Linear regression of C⍺RMSD values demonstrated no correlation with any of the dependent variables (all R < 0.3, Supplementary Figures 1–3). A similar analysis was performed using the difference in Rosetta total score in Rosetta energy units (REU) between the Acd mutant and WT simulations and again no correlation between REU and the dependent variables was observed (all R < 0.3, Supplementary Figures 4–6). This confirmed that traditional analyses such as RMSD and changes in total energy are insufficient in predicting these phenomena, as previously observed^30,31^.

### Energetic components support descriptive modeling

Next, we analyzed the correlations between Rosetta score deltas and the values from the experimental dataset and attempted to describe the system through linear regression. We observed that many of the score delta features were individually more correlative than any of the structure-independent bioinformatics terms analyzed in our previous efforts (Supplementary Tables 6 and 7)^10^. Table 1 displays the ten features from the Rosetta score function that are most correlative with Acd mutant protein soluble fraction. We identified that the most correlative terms were energetic changes at the Acd incorporation site, demonstrating the importance of our structural modeling. Given the correlations of the independent Rosetta score terms, we constructed a set of multiple linear regressions (MLRs) in which we performed backwards selection to arrive at a small number of features which strongly describe the dependent variables. Table 2 details the elements of the MLRs including the feature set, dependent variable, number of model features, R, and ƒ statistics for the models. The MLR analyses convey the ability for small numbers of Rosetta derived features to describe each protein subset for all three dependent variables above an R of 0.725. Additionally, we observed that unlike our prior study where the most predictive terms (protein domain and secondary structure) were not capable of being generally applied to both protein datasets, these MLRs are capable of effectively describing Acd mutant protein soluble yield, total yield, and soluble fraction in the combined dataset (Table 2).

**Table 1:** The most correlative Rosetta energy features with Uaa mutant soluble fraction.

Top features	RCSF	R value	Description
1	rama_prepro_8A	0.500	Energy of backbone phi and psi angles
2	fa_atr_Site	0.486	Attractive energy of inter-residue atoms
3	residue_total_score_Site	0.434	Linear combination of score function energies
4	fa_intra_atr_xover_Site	0.422	Attractive energy of intra-residue atoms
5	hbond_sr_bb_Site	0.349	Short-range hydrogen bond energies
6	fa_rep_Site	0.336	Repulsive energy of inter-residue atoms
7	lk_ball_iso_Site	0.334	Isotropic contribution to Solvation
8	hbond_sc_Site	0.328	Sidechain hydrogen bond energies
9	lk_ball_iso_8A	0.322	Isotropic contribution to Solvation
10	fa_intra_atr_xover_8A	0.311	Attractive energy of intra-residue atoms

The suffixes of _Site and _8A correspond to energies at the mutation site and the 8A contacting sphere respectively.

**Table 2:** Summary statistics of RCSF multiple linear regressions.

MLR	R	Adj. R	F statistic	Prob. F statistic	Number features
Soluble yield RCSF	0.899	0.872	16.86	2.15E−11	10
Total yield RCSF	0.947	0.940	77.20	6.21E−21	5
Soluble fraction RCSF	0.725	0.670	6.817	1.86E−05	7

### Detailed structural analysis provides basis for correlation

Following our investigation of Rosetta features, we performed the same analyses for a set of ESTs, to determine if more generalizable terms could be used in a similar approach. Although the structure-independent terms were unable to achieve a Pearson correlation above 0.25, the new contact-based ESTs were able to achieve correlations up to R values of 0.503. Table 3 displays the ten ESTs that are most correlative with Acd mutant protein soluble fraction. Interestingly, we observed that the most correlative terms directly report on changes in contacts due to Acd incorporation. These results closely match the most correlative Rosetta terms as they also reported largely on the Acd mutation site. Moreover, we observed that EST MLRs (Table 4) were able to describe the soluble yield and soluble fraction datasets similarly to the Rosetta terms MLRs (Table 2), but were significantly less correlative with the total yield dataset. Overall, we were highly encouraged that this approach might be generalizable beyond the use of Rosetta-specific score terms based on the correlations of the contact and bioinformatics-based terms computed from the PyRosetta generated mutant structures.

**Table 3:** The most correlative EST features with Uaa mutant soluble fraction.

Top features	EST	R Value	Description
1	np_bb_sc_intra	0.503	Intra-residue backbone to sidechain nonpolar contacts
2	total_np_contacts	0.488	Total number of nonpolar contacts
3	np_sc_sc_inter	0.390	Inter-residue sidechain to sidechain nonpolar contacts
4	total_contacts	0.376	Total number of polar and nonpolar contacts
5	p_sc_sc_inter	0.321	Inter-residue sidechain to sidechain polar contacts
6	ASA	0.241	Accessible surface area
7	kD_cyclohexane_water	0.226	Measure of hydrophobicity
8	RSA	0.223	Relative accessible surface area
9	kD_vapor_to_water	0.219	Measure of hydrophobicity
10	kD_octanol_to_water	0.215	Measure of hydrophobicity

Definitions of features can be found in Supplementary Table 3.

**Table 4:** Summary statistics of ESF multiple linear regressions.

MLR	R	Adj. R	F statistic	Prob. F statistic	Number features
Soluble yield ESF	0.903	0.794	4.427	2.06E−05	10
Total yield ESF	0.738	0.704	10.78	7.42E−07	5
Soluble fraction ESF	0.708	0.649	6.189	4.86E−05	7

### RCSF and ESF features produce accurate classifiers

Since our Rosetta and EST sets were significantly more correlated with soluble fraction over the previously explored structure-independent bioinformatics terms, we next focused on assessing the maximal utility of these terms by attempting to classify positional tolerance of Acd mutation based on prediction of soluble fraction. Since the number of potential features is larger than the dataset, we reduced dimensionality through feature selection with the SelectKBest module in scikit-learn. An upper threshold of 10 features was set to avoid overfitting. Furthermore, we were interested in understanding which ML methods provide the most predictive power for each experimental value with these features, so we tested a wide variety of algorithms. Feature selection coupled with untuned model prediction showed varying results for the optimal number of features and those that were selected for each classification task can be found in Supplementary Tables 8 and 9.

**Figure 2:**
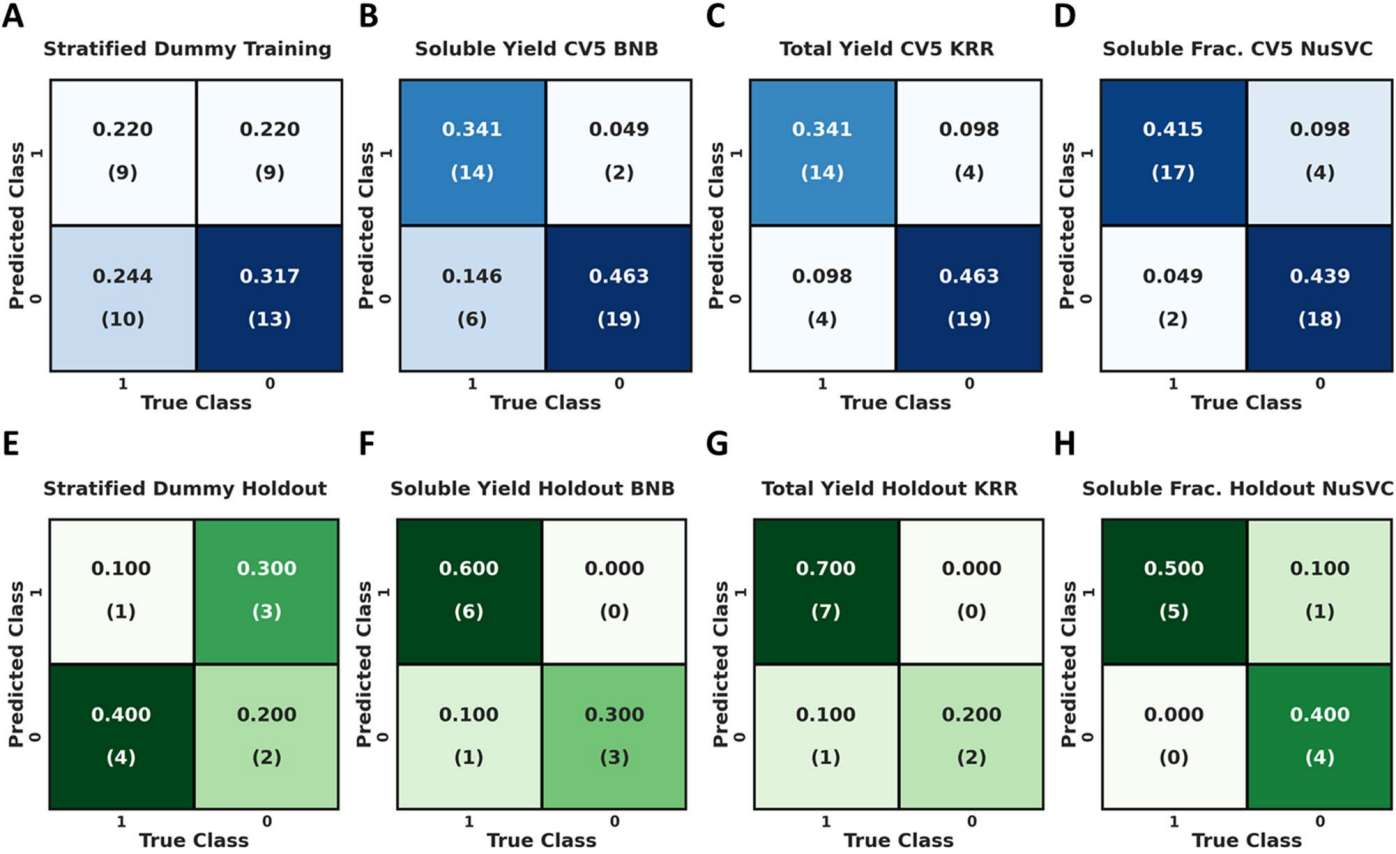
Confusion matrices showing predictions from stratified CV5 and prediction of the holdout. For a binary classifier the top left represents true positives, the top right represents false positives, the bottom left represents false negatives, and finally the bottom right represents true negatives. The top row (**A**–**D**), shows cross validation scores for RCSFs and the bottom row (**E**–**H**), shows holdout prediction for RCSFs. Matrices A and E display the results of a dummy classifier using the stratified criterion, matrices B and F display the tuned soluble yield models, matrices C and G display the tuned total yield models, and matrices D and H display the tuned soluble fraction models. Note: BNB, KRR, and NuSVC are the Bernoulli Naïve Bayes, Kernel Ridge Regression, and Nu Support Vector classifiers respectively. Advanced metrics can be found in Table 5.

Following feature selection, each feature selected ML model was tuned using exhaustive grid searching (stratified CV5) to identify the optimal hyperparameters for the soluble yield, total yield, and soluble fraction models for both feature sets. First, we focused on generating RCSFs from Rosetta score terms and analyzed confusion matrices (Fig. 2) for RCSF cross validation and holdout prediction across every dependent variable. Additionally, a dummy classifier is presented for a baseline comparison (Fig. 2A), which performed as expected given the stratified criterion with a prediction training accuracy of ~ 53% and training precision of ~ 50%. The soluble yield RCSF (Fig. 2B) demonstrated a training accuracy of ~ 81% with a precision of ~ 88%. Very similarly, the total yield RCSF (Fig. 2C) was predicted at ~ 81% accuracy, but with a slightly lower precision of ~ 78%. Lastly, our soluble fraction RCSF (Fig. 2D), predicted with a training accuracy 85.4% and precision of ~ 81%.

The ability of the RCSFs to serve as practical tools for prediction of Acd mutant protein yield and solubility requires accurate prediction of never-before-seen data. Again, for comparison to random classification, a dummy classifier (Fig. 2E) is shown which predicted the holdout with an accuracy of 30% with 20% precision. Figure 2E–H show the confusion matrices for the prediction of the holdout datasets for every dependent variable. Here, both the soluble and total yield RCSFs (Fig. 2F,G) demonstrated 90% holdout accuracy and perfect precision. The soluble fraction RCSF (Fig. 2H), predicted the holdout at 90% accuracy with ~ 83% precision.

**Figure 3:**
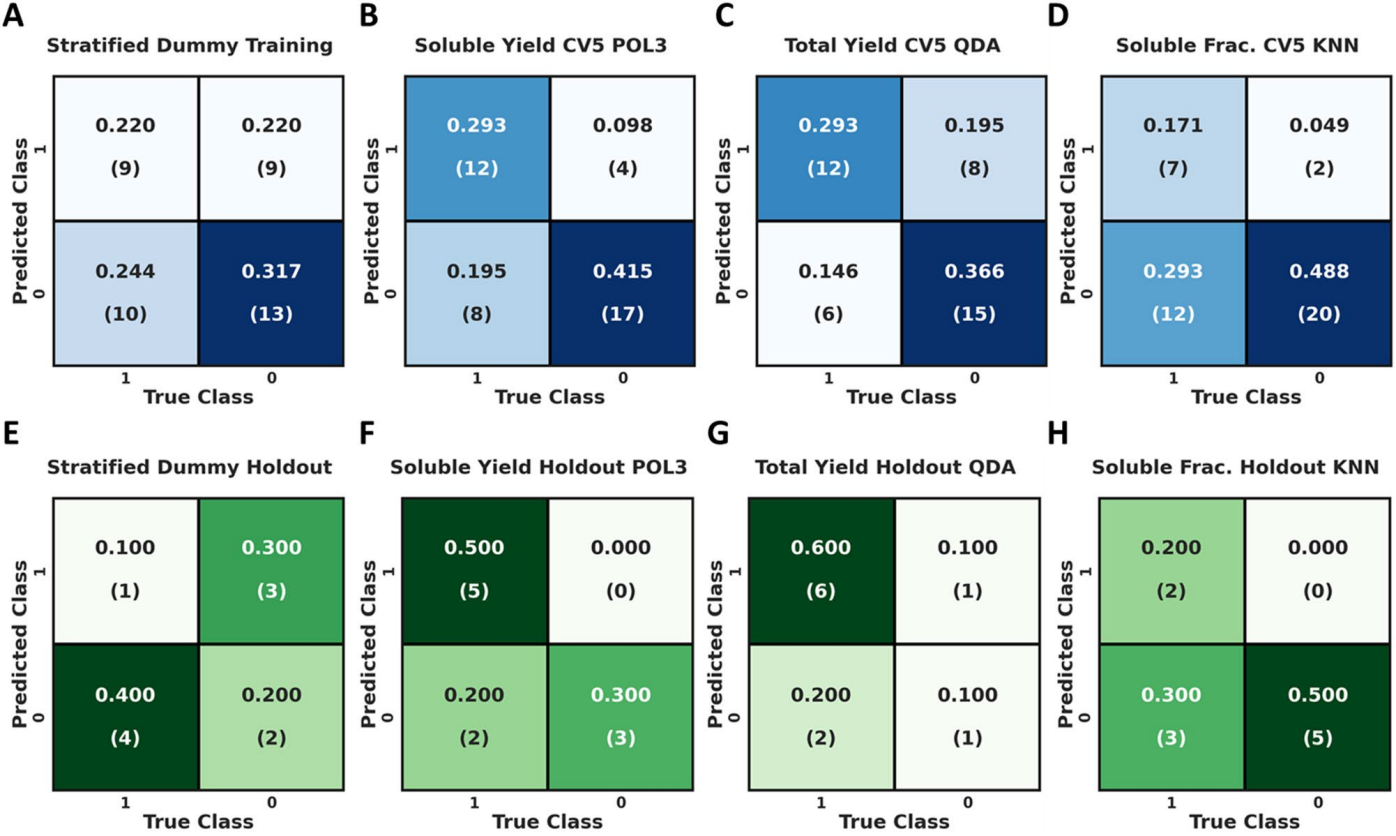
Confusion matrices showing predictions from stratified CV5 and prediction of the holdout. The top row (**A**–**D**), shows cross validation scores for ESFs and the bottom row (**E**–**H**), shows holdout prediction for ECSFs. Matrices A and E display the results of a dummy classifier using the stratified criterion, matrices B and F display the tuned soluble yield models, matrices C and G display the tuned total yield models, and matrices D and H display the tuned soluble fraction models. Note: POL3, QDA, and KNN are the Support Vector Degree 3, Quadratic Discriminant, Analysis, and K Nearest Neighbors classifiers respectively. Advanced metrics can be found in Table 5.

To confirm the generalizability of generating predictive machine learned score functions from sets of correlative terms, we created an identical set of ESFs from the ESTs. Similarly to the RCSF analysis, Fig. 3A–D displays confusion matrices for the ESF cross validation and holdout prediction across every dependent variable, along with dummy classifier metrics. The soluble yield ESF (Fig. 3B) demonstrated a training accuracy of ~ 71% with a precision of 75%. The total yield ESF (Fig. 3C) predicts at ~ 66% accuracy, but with a low precision of 60%. Additionally, our soluble fraction ESF (Fig. 3D), demonstrated a training accuracy ~ 66% and precision of ~ 78%. Moreover, analysis of the confusion matrices for the prediction of the holdout datasets of the dummy classifier (Fig. 3E) and ESFs (Fig. 3F–H) demonstrated that the ESFs performed similarly, albeit slightly less effectively than the RCSFs. The soluble yield ESFs (Fig. 3F) demonstrated 80% holdout accuracy and perfect precision, while the total yield ESF (Fig. 3G) and the soluble fraction ESF (Fig. 3H), both predicted the holdout at 70% accuracy, with 85.7% and 100% precision respectively. Table 5 displays a unified table of classification statistics for RCSFs and ESFs across all the dependent variables.

**Table 5:** Classification metrics of classifiers.

Metric	Soluble yield best	Total yield best	Soluble fraction best
**RCSF**			
CV accuracy	0.805	0.805	0.854
Holdout accuracy	0.900	0.900	0.900
CV5 precision	0.875	0.777	0.810
Holdout precision	1.000	1.000	0.833
CV5 recall	0.700	0.777	0.895
Holdout recall	0.857	0.875	1.000
CV5 F1 score	0.778	0.777	0.850
Holdout F1 score	0.923	0.933	0.909
**ESF**			
CV accuracy	0.707	0.659	0.659
Holdout accuracy	0.800	0.700	0.700
CV5 precision	0.750	0.600	0.778
Holdout precision	1.000	0.857	1.000
CV5 recall	0.600	0.667	0.368
Holdout recall	0.714	0.750	0.400
CV5 F1 score	0.667	0.632	0.500
Holdout F1 score	0.833	0.800	0.571

CV5 corresponds to Stratified fivefold cross validation.

### Structural accommodation and desolvation of Acd convey predictivity

**Figure 4:**
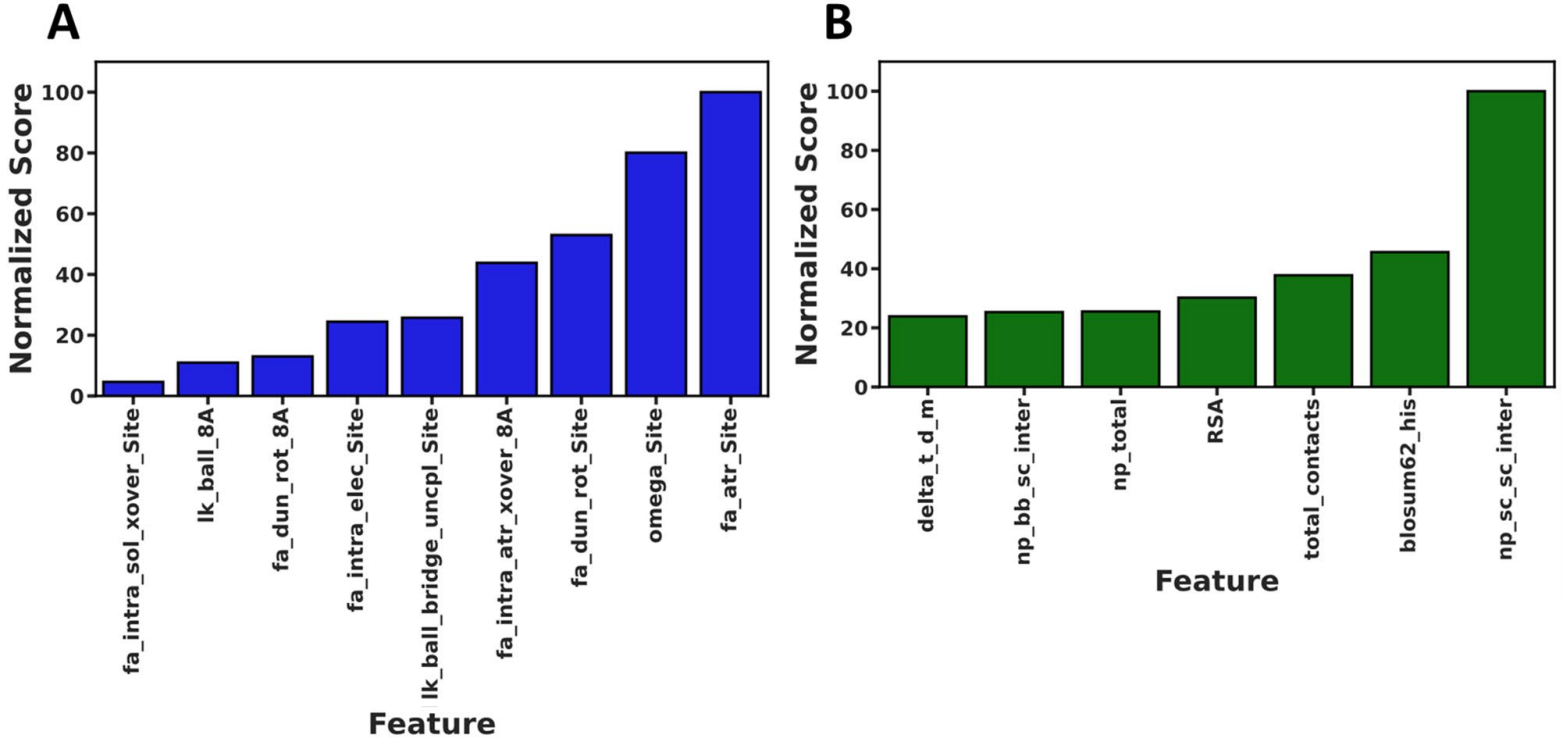
Normalized model feature importance from soluble fraction Logistic RCSF (**A**) and Logistic ESF (**B**). The most important feature has score 100 and each score less than 100 is used at that percent of the most important feature.

Finally, after demonstrating that RCSFs and ESFs can be used to accurately classify Acd mutant protein soluble fraction, we focused on identifying which features were responsible for generating this predictive accuracy. Since extraction of model feature importance for nonlinear algorithms other than decision tree-based methods is not readily available in scikit-learn, we performed model feature importance analyses on LOG models (Fig. 4, Supplementary Tables 12–15). Analysis of the feature importance in the soluble fraction RCSF LOG model demonstrated that the most important Rosetta score terms were fa_atr_Site, omega_Site, fa_dun_rot_Site, fa_intra_atr_xover_8A, lk_ball_bridge_uncpl_Site, and fa_intra_elec_Site (Fig. 4A). These terms represent the energies associated with pairwise van der Waals attraction, the Acd residue specific backbone omega dihedral angle and Acd rotameric preferences, the intra-residue van der Waals attraction of the contacting sphere, the uncoupled bridging contribution of the Lazaridis-Karplus solvation of Acd, and the intra-residue electrostatic energy of Acd respectively. The remaining selected terms corresponding to fa_dun_rot_8A, lk_ball_8A, and fa_intra_sol_Site were used to a significantly lesser extent than the most import feature (< 10% of fa_atr) and correspond to the internal energy of the sidechain from Dunbrack’s statistics of residues in the contact sphere, the anisotropic contribution of the Lazaridis-Karplus solution of the contact sphere and intra-residue solvation for the Acd site.

Analysis of the feature importance in the soluble fraction ESF LOG model demonstrated that all the selected features were similarly important, except for np_sc_sc_inter which had an increased importance. The remaining features were blosum62_his, total_contacts, RSA, np_total, np_bb_sc_inter, and delta_t_d_m (all terms detailed in Supplementary Table 3). The majority of these terms (np_sc_sc_inter, total_contacts, np_total, and np_bb_sc_inter) are nonpolar contacts computed between the Acd and the surrounding residues derived from our PyRosetta modeling. RSA is the relative accessible surface area of the residue which is to be mutated to Acd and are also a function of the residue’s contacts^39^. The blosum62_his and delta_t_d_m are the BLOSUM values associated with the mutation site residue when mutated to histidine, and a measure of the change in peptide meting temperature vs glycine^40,41^. As was observed during feature correlation analysis, the selected RCSF and ESF features are similar and represent properties associated with the ability of a protein to accommodate the large aromatic sidechain of Acd.

## Discussion

Our identification of Rosetta score terms and ESTs as correlative features with Acd incorporation tolerability based on soluble fraction and the combination of these terms through machine learning to generate RCSFs and ESFs has several key advantages over the methods previously employed. Previously, we hypothesized that positions which had low soluble fractions (Fig. 1B, amino acids colored in red) would show greater structural deviations between the different local relax simulations in the Acd mutant simulations. Additionally, we expected that the Rosetta total score would reflect structural perturbation induced by Acd incorporation. Although we did not observe correlations between Acd mutant protein soluble fraction and either the global structural deviations or the total energy computed, we did see striking correlations for local physical interactions and energies associated with perturbations at and around the mutation site. These observations are consistent with our previous analyses predicting the change in energy of mutations at protein–protein interfaces and positions in peptides that imbue proteolytic resistance upon backbone thioamidation^30,31^. Moreover, this phenomenon was reflected in the ESTs as they too demonstrated that decomposed features were more correlative than their total feature counterparts (i.e. number of sidechain-sidechain nonpolar contacts rather than total number of contacts). Additionally, we observed that ESTs computed from our structural models were more correlative than the structure independent bioinformatics terms, supporting the fact that predictivity is rooted in the local structural changes more generally, and is not just attributable to Rosetta energies. The generalizability of this approach overall can also be supported by others who have used energy-based machine learning methods that are not RCSFs^42–44^. For example, Adeshina et al*.* minimized protein ligand complexes with Rosetta and used a subset of energies along side other features in an effort to reduce the false positive rate in ligand virtual screening^42^. Outside of the Rosetta energy function, Rauer et al. simulated molecules in various solvents using MD in order to predict hydration energies^43^. Taken together, these studies along with our investigations demonstrate the strong predictivity of energy-based and empirical ML models and support the idea that many different computational platforms can likely be used to generate predictions about Uaa incorporation if ML is applied to perturbation of local structural features.

Beyond delivering significantly improved predictive capacity, the structure-based features from this investigation were able to describe the important properties of each site as related to Acd tolerance. This is intriguing as it begins to uncover the mechanisms behind the clear differences in total yield, soluble yield, and soluble fraction observed, even when attempting to make conservative mutations (i.e. Phe-to-Acd) or in mutating the same residue at different sites (i.e. LexA Phe 12 vs. LexA Phe 111). Consulting chemical intuition, we would hypothesize that positions which tolerate mutation to Acd would need to accommodate backbone and sidechain rotameric states capable of desolvating the bulky, aromatic Acd side chain. If they are incapable of doing so, the Acd side chain will be undesirably exposed to solvent or forced to clash with other residues. Indeed, this chemical intuition informed our previous attempts to determine correlations to individual properties^10^, and although these were not able to predict tolerability, they are nonetheless related to the top RCSF and ESF features. Many of the top ESF features correspond to hydrophobic contacts, solvent accessible surface area, and measures of hydrophobicity. At the same time, the top RCSF features correspond to van der Waals energies, peptide backbone angle preferences, and solvation energies. If we consider an example of mutation of Phe to Acd at position 12 (tolerated) versus at 111 (not tolerated), we can observe these features in action. At position 12, Acd is buried and adopts a clash free conformation. At position 111, while Acd is fully desolvated, it is too large and highly clashed with surrounding residues. A different example. where simply considering the identities of the native residues would have led to incorrect predictions of tolerance but our models allow accurate classification are Ser60 and Tyr98. Naively, one would expect a Tyr to Acd mutation to be better tolerated than a Ser to Acd mutation since Tyr is a bulky aromatic (hydrophobic) residue like Acd, and Ser is much smaller and considered to be polar. In this specific example however, the Ser mutation is tolerated, while the Tyr mutation is not. Fortunately, our models could accurately differentiate these two positions and inspection of the modeled structures allows chemical intuition to match the ESF and RCSF predictions. While position is 60 is solvent exposed, the Acd side chain is able to form many hydrophobic interactions and fill a small cleft. Position 98 is found at the dimer interface, and the Acd sidechain induces steric clashes due to its greater size than Tyr. These examples are rewarding, as they match our chemical intuition, demonstrating that this method provides models with a rationalizable basis for prediction as previously observed in our investigation of modified peptides^30^.

Comparison of the utilities of the RCSFs and ESFs specifically can be made based on training and holdout performance. Rewardingly, for all of our models, we observed only minor differences in the quality of the holdout prediction as compared to the training albeit with different predictive powers. Across the board, our RCSFs displayed training accuracies, precisions, and recalls routinely above 80% and translatability of those predictive capacities to the holdout. These data indicate good generalizability to new LexA and RecA data and show a strong ability to select for positives, which would tremendously enrich small scale screens for tolerated sites over the unbiased experimental methods described above. Our ESF models were demonstrably weaker predictors (training and holdout accuracies, precisions, recalls of 60–70%) than the RCSFs, but do show enrichment versus the dummy classifiers and translatability to the holdout, supporting the utility of their features.

With regards to model effectiveness in new protein systems, since we are using score deltas that are intrinsically normalized to the native structure, and the observed accurate testing on a diverse holdout set (sites with various protein primary, secondary, and tertiary structures) support the use of our models in predicting Acd tolerability in other systems. For other unique proteins, if the energy features computed from PyRosetta simulations fall within the distributions of our feature vectors laid out in Supplementary Table 4, these models may also demonstrate utility. Nonetheless, the facile method described herein along with our previous two studies using RCSFs, provide strong evidence that the construction of custom scoring functions for prediction of a specific phenomenon is a superior strategy compared to the development of a singular generalized scoring function (forcefield) for a Uaa such as Acd. Ultimately, this investigation demonstrates that we have uncovered a method for predicting current datasets, suggesting that construction of a dataset that includes both different Uaas and multiple proteins may yield a generally predictive system of interest to the field.

## Conclusion

Prior efforts to predict the parameters which reveal the tolerability of mutations to Uaas have been limited and thus far unsuccessful, leading researchers to use empirical methods. Herein, we focused on demonstrating that features, rooted in local structure computed from PyRosetta simulations, can serve as a basis for the development of predictive ML models. Uaa protein mutants of interest were simulated using PyRosetta yielding structural models which can be used to train RCSFs and ESFs that, for the first time, accurately predict Acd mutant protein soluble and total yield as well as soluble fraction with high accuracy. Given our recent development of Acd as a probe for imaging in living mammalian cells, we are excited about using the approach described here to train models for predicting well-tolerated labeling sites for imaging applications. The success of these models also has broad implications for the Uaa community and more generally for those interested in predicting biological phenomena via computation methods. The observed high cross validation scores, as well as generalizability, exemplified by accurate prediction of a diverse well-balanced holdout dataset, demonstrate that this modeling approach can identify key features for highly abstract experimental parameters in even small subsets of data. In the long term, we will continue to investigate the ability for RCSFs and ESFs to be used in conjunction with each other and additional features. Lastly, we have made our models for prediction of novel Acd mutant protein data available on our GitHub.

## Limitations and outlook

Although this methodology demonstrates that RCSFs and ESFs can accurately predict biological phenomena which elude more traditional approaches, the current study was performed on a small dataset (51 datapoints). We used a standard holdout percentage of 20%, corresponding to a low overall number of datapoints (10) for validation on never-before-seen data. It is likely that the models trained here are not generalizable beyond Acd and the LexA and RecA proteins, as this dataset is not expected to capture the diversity of protein structures across the proteome and other Uaas would have physical properties that are distinct from those of Acd so the relevant features for those Uaas were not selected here. Moreover, we encourage others adopting the RCSF method to consider the applicability of the Rosetta score function used for running simulations. For example, the betaNov16 score function used here has been updated for improved ligand docking as RosettaGenFF/beta_genpot. While this change would not be expected to affect our results since no ligands were present, those attempting to perform similar studies in the presence of ligands should evaluate the currently available Rosetta score functions and select the appropriate score function depending on the task. Despite these limitations, the results herein and in prior reports demonstrate that RCSFs and ESFs are highly useful for producing interpretable ML models for predicting complex biological phenomena^30,31^.

## Supplementary Information

Supplementary Information.

## Data Availability

The datasets and analyses generated in the current study are included in this article as well as the Supplementary Information and are available from the corresponding authors on reasonable request. Codes have been made available on our lab GitHub at https://github.com/ejp-lab/EJPLab_Computational_Projects/tree/master/RML_ACD.
