# Supplementary material for: Biomolecular simulation based machine learning models accurately predict sites of tolerability to the unnatural amino acid acridonylalanine
Source: Sci Rep. 2021 Sep 15;11:18406. doi: 10.1038/s41598-021-97965-2 (PMC8443755; doi:10.1038/s41598-021-97965-2)
Supplement: Supplementary file 1 — Supplementary Information. [file 41598_2021_97965_MOESM1_ESM.pdf]

**Supplementary Information**

# Biomolecular Simulation Based Machine Learning Models Accurately Predict Sites of Tolerability to the Unnatural Amino Acid Acridonylalanine

Sam Giannakoulias,[‡a] Sumant R. Shringari,[a] John J. Ferrie,[*,b] and E. James Petersson*[a]

[a]Department of Chemistry, University of Pennsylvania, Philadelphia, Pennsylvania 19104, USA
[b]Department of Molecular & Cell Biology, University of California, Berkeley, Berkeley, California, 94720, United States

*Email: JJF: jferrie@berkeley.edu; EJP: ejpetersson@sas.upenn.edu

## Table of Contents:

1. **Software**……………………………………………………………………………….. **S02**
2. **Datasets**………………………………………………………………………………... **S02**
3. **Definitions**……………………………………………………………………………... **S04**
4. **Starting Structures** …………………………………………………………………… **S04**
5. **Acridon-2-ylalanine (Acd) Modeling** ………………………………………………… **S05**
6. **Feature Analysis**……………………………………………………………………… **S10**
7. **Descriptive Analysis**…………………………………………………………………… **S12**
8. **Dimensionality Reduction**……………………………………………………………… **S14**
9. **Tuning Parameters**……………………………………………………………………… **S22**
10. **Feature Importance**…………………………………………………………………… **S23**
11. **References**……………………………………………………………………………… **S25**

## Software

Models which predict the effect on yield or solubility of Acd containing proteins required the following software: python version 3.7, PyRosetta, and the following packages: numpy, scipy, pandas, scikit-learn[1], and seaborn. Instruction on how the models trained in this work can be applied to novel proteins or positions can be found on our GitHub at https://github.com/ejp-lab/EJPLab_Computational_Projects/tree/master/RML_ACD. An Anaconda yml file is freely available for download and will create a virtual environment suitable for this program.

## Datasets

Datasets used to train the models in this work were acquired in a previously published study by the Petersson and Kohli labs.[2] The Acd incorporation data spans 51 residue positions across the LexA and RecA proteins. A large well-balanced holdout dataset for validating machine learning (ML) models was curated by randomly selecting equal amounts of LexA and RecA data and ensuring that the data points closely matched the distribution of the soluble fraction data. The training and holdout data sets can be found on our github at https://github.com/ejp-lab/EJPLab_Computational_Projects/tree/master/RML_ACD/Dataset.

**Supplementary Table 1.** Members of the holdout dataset

| Data Set | Position Number | Native Residue | Sol Yield (nM) | Tot Yield (nM) | Sol Frac (%) | Sol Yield Class | Tot Yield Class | Sol Frac Class |
|---|---|---|---|---|---|---|---|---|
| Training | LexA 9 | GLN | 350 | 720 | **49** | 0 | 0 | **1** |
| Training | LexA 12 | PHE | 870 | 1300 | **65** | 1 | 0 | **1** |
| Training | LexA 21 | GLN | 760 | 1400 | **56** | 1 | 0 | **1** |
| Training | LexA 22 | THR | 1000 | 1600 | **64** | 1 | 1 | **1** |
| Training | LexA 33 | GLN | 670 | 1800 | **38** | 1 | 1 | **0** |
| Training | LexA 36 | GLY | 520 | 1400 | **37** | 1 | 0 | **0** |
| Training | LexA 37 | PHE | 910 | 1600 | **57** | 1 | 1 | **1** |
| Training | LexA 60 | SER | 1300 | 1900 | **68** | 1 | 1 | **1** |
| Training | LexA 74 | GLU | 820 | 1400 | **61** | 1 | 0 | **1** |
| Training | LexA 81 | ARG | 680 | 2500 | **27** | 1 | 1 | **0** |
| Training | LexA 97 | HIS | 510 | 840 | **61** | 0 | 0 | **1** |
| Training | LexA 98 | TYR | 66 | 960 | **7** | 0 | 0 | **0** |
| Training | LexA 104 | LEU | 75 | 960 | **8** | 0 | 0 | **0** |
| Training | LexA 106 | LYS | 220 | 910 | **24** | 0 | 0 | **0** |
| Training | LexA 108 | ASN | 190 | 1100 | **17** | 0 | 0 | **0** |
| Training | LexA 111 | PHE | 0 | 750 | **0** | 0 | 0 | **0** |
| Training | LexA 130 | LEU | 24 | 1300 | **2** | 0 | 0 | **0** |
| Training | LexA 135 | THR | 280 | 2000 | **13** | 0 | 1 | **0** |
| Training | LexA 138 | ASP | 420 | 2200 | **19** | 0 | 1 | **0** |
| Training | LexA 150 | ASP | 5 | 1700 | **0** | 0 | 1 | **0** |
| Training | LexA 161 | GLN | 360 | 1300 | **28** | 0 | 0 | **0** |
| Training | LexA 162 | GLY | 39 | 1400 | **3** | 0 | 0 | **0** |
| Training | LexA 166 | GLU | 240 | 970 | **25** | 0 | 0 | **0** |
| Training | LexA 172 | SER | 130 | 1300 | **10** | 0 | 0 | **0** |
| Training | LexA 174 | PHE | 140 | 1100 | **13** | 0 | 0 | **0** |
| Training | LexA 178 | VAL | 200 | 1300 | **15** | 0 | 0 | **0** |
| Training | LexA 186 | PHE | 140 | 1500 | **5** | 0 | 0 | **0** |
| Training | RecA 4 | GLU | 2400 | 9700 | **25** | 1 | 1 | **0** |
| Training | RecA 33 | ARG | 7400 | 7400 | **100** | 1 | 1 | **1** |
| Training | RecA 65 | TYR | 3700 | 6300 | **60** | 1 | 1 | **1** |
| Training | RecA 86 | GLU | 5400 | 6600 | **81** | 1 | 1 | **1** |
| Training | RecA 102 | ILE | 2700 | 4000 | **67** | 1 | 1 | **1** |
| Training | RecA 124 | GLN | 6000 | 7400 | **81** | 1 | 1 | **1** |
| Training | RecA 134 | ARG | 2500 | 4500 | **56** | 1 | 1 | **1** |
| Training | RecA 156 | GLU | 3400 | 6200 | **54** | 1 | 1 | **1** |
| Training | RecA 197 | MET | 5400 | 5800 | **93** | 1 | 1 | **1** |
| Training | RecA 206 | PRO | 4400 | 6000 | **73** | 1 | 1 | **1** |
| Training | RecA 233 | GLU | 770 | 1200 | **66** | 1 | 0 | **1** |
| Training | RecA 266 | GLU | 220 | 4100 | **5** | 0 | 1 | **0** |
| Training | RecA 277 | LEU | 2200 | 4900 | **45** | 1 | 1 | **1** |
| Training | RecA 321 | LYS | 1400 | 5400 | **26** | 1 | 1 | **0** |
| | | | | | | | | |
| Holdout | LexA 6 | ALA | 1300 | 1900 | **68** | 1 | 1 | **1** |
| Holdout | LexA 86 | GLU | 720 | 3200 | **22** | 1 | 1 | **0** |
| Holdout | LexA 105 | PHE | 2.3 | 1200 | **2** | 0 | 0 | **0** |
| Holdout | LexA 140 | ARG | 340 | 2100 | **16** | 0 | 1 | **0** |
| Holdout | LexA 183 | GLN | 150 | 1300 | **18** | 0 | 0 | **0** |
| Holdout | RecA 85 | ARG | 6100 | 7200 | **86** | 1 | 1 | **1** |
| Holdout | RecA 121 | THR | 5500 | 7000 | **79** | 1 | 1 | **1** |
| Holdout | RecA 150 | THR | 2100 | 5800 | **36** | 1 | 1 | **0** |
| Holdout | RecA 213 | ASN | 2500 | 5200 | **47** | 1 | 1 | **1** |
| Holdout | RecA 311 | ASP | 1600 | 3700 | **44** | 1 | 1 | **1** |

**Supplementary Table 1.** Experimental data of the holdout dataset. The classification cutoffs were as follows: Soluble Yield (Sol Yield) set at 520 nM, balancing actives and inactives at 53% and 47%, respectively; Total Yield (Tot Yield) set at 1600 nM, balancing actives and inactives at 51% and 49%, respectively; Soluble Fraction (Sol Frac) set at 39%, balancing actives and inactives at 47% and 53%, respectively.

## Definitions

The following table represents the abbreviations used within the rest of the document.

**Supplementary Table 2.** Abbreviations

| Abbreviation | Full Name |
|---|---|
| Acd | Acridon-2-ylalanine |
| LOG | Logistic Classifier |
| KRR | Ridge Classifier |
| LDA | Linear Discriminant Analysis Classifier |
| SVC | Support Vector Classifier |
| KNN | K Nearest Neighbors Classifier |
| BNB | Bernoulli Naïve Bayes Classifier |
| GPC | Gaussian Process Classifier |
| PASSAG | Passive Aggressive Classifier |
| RBF | Radial Base Function Kernel |
| SIG | Sigmoid Kernel |
| LIN | Linear Kernel |
| POL2 | Polynomial Degree 2 Kernel |
| POL3 | Polynomial Degree 3 Kernel |
| Adj. | Adjusted |
| Prob | Probability |

## Starting Structures

The crystallographic LexA structure (PDB: 1JHH[3]) is a dimer where the N-terminal domain of the second monomeric unit is missing due to incomplete electron density. Additionally, the C-terminal domain of the second monomeric unit has missing electron density in a loop corresponding to residues 87-92 (sequence LLAQQH). Finally, the three C-terminal residues of the second monomeric unit (sequence DWL) are missing due to insufficient electron density. In order to prepare a full model of the LexA homodimer, the C-terminal domain of the second monomeric was saved it as its own file where loop remodeling was performed on the two missing regions to complete the domain using Rosetta Kinematic Closure (KIC). To model the N-terminal domain, we used PyMOL to align the complete first monomeric unit to the loop remodeled C-terminal domain of the second monomeric unit. The complete monomer which was now in the mirrored orientation was saved as its own file. We finally opened a new PyMOL session with both

the entire first monomeric unit as well as the aligned mirrored full monomeric unit. These two files were saved as a joint object producing the complete homodimer.

The RecA structure (PDB: 3CMW[4]) is originally a polymer which is bound with DNA and other small molecules. For simplicity in modeling, the original RecA structure was trimmed to a single monomeric unit without any DNA present. Both the LexA and RecA structures were relaxed after initial pre-processing to be used in mutational modeling. The structures were relaxed with a Cartesian fast-relax in PyRosetta with the following options: a MoveMap set for both backbone and sidechain minimization, the beta_nov_16_cart score function, lbfgs_armijo_nonmonotone minimizer, and the minimize_bond_angles flag set to True.

**Acridon-2-ylalanine (Acd) Modeling**

In order to mutate sites of interest to Acd, we used the same params and rotamer library files generated in our previous work.[2] These requisite files were generated according to the protocol in Renfrew *et al.*[5]

To mutate a residue to Acd, PyRosetta must be initialized with the following extra options:

```
from pyrosetta import *
init(extra_options='-extra_res_fa acd.params)

pose = pose_from_pdb('PDB_NAME')

mutator = pyrosetta.rosetta.protocols.simple_moves.MutateResidue(res1_num, res1_name')
mutator.apply(pose)
```

Following each mutation to include Acd, all complexes were "locally" relaxed with five independent simulations and the following parameters: the beta_nov16 score function, a MoveMap set for both backbone and sidechain optimization of only residues that have a Cα atom within 8 Å of the Cα of the Acd residue, dualspace and minimize_bond_angles set to True, and the

lbfgs_armijo_nonmonotone minimizer. For each mutation site, separate local relaxes were performed for the wildtype (WT) control to enable direct comparison to the corresponding locally relaxed residues in Acd simulations.

RMSD analysis of the five local relax simulations for the Acd and WT proteins demonstrated a convergence to a single resultant structure as the highest average RMSD difference observed for any structure was less than 1.4 Å and the majority are substantially below an RMSD of 1 Å. Note that the only residues considered in the RMSD were those for which the MoveMap allowed movement in the local relax (8 Å sphere).

RMSD analysis of the lowest energy Acd structure with respect to the lowest energy WT structure for each residue in the dataset demonstrated a more mixed set of results. Many showed only small deviations in structure as a result of Acd incorporation, while some positions showed deviations up to 4.073 Å.

Supplementary Figures 1-6 represent attempts to correlate Rosetta simulation with Acd yield and solubility data. Firstly, we investigated average RMSD differences between the Acd and WT simulations. Supplementary Figures 1-3 demonstrate that there is no correlation between RMSD and any of the three dependent variables (soluble yield, total yield, soluble fraction). We also investigated the difference in Rosetta total score between the Acd and WT simulations. We observed yet again that there is no correlation between Rosetta REU and our data.

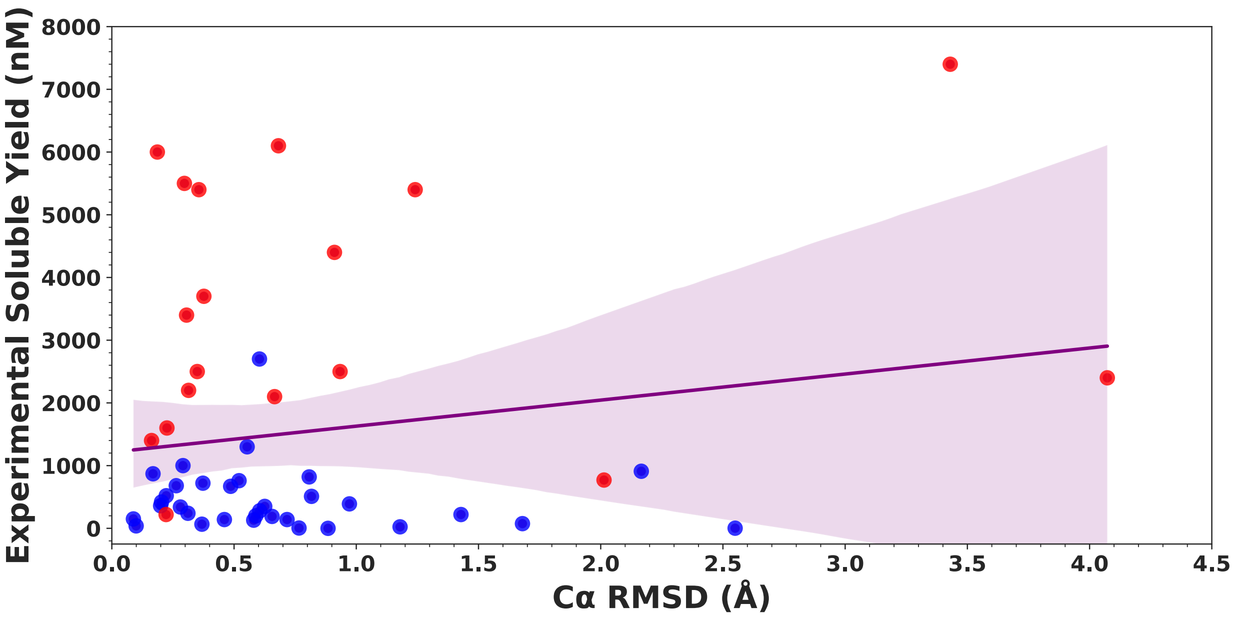


**Supplementary Figure 1.** Plot demonstrating the RMSD distribution between Acd and WT simulations vs experimental soluble yield data. Points for RecA data are colored red and points for LexA data are colored blue.

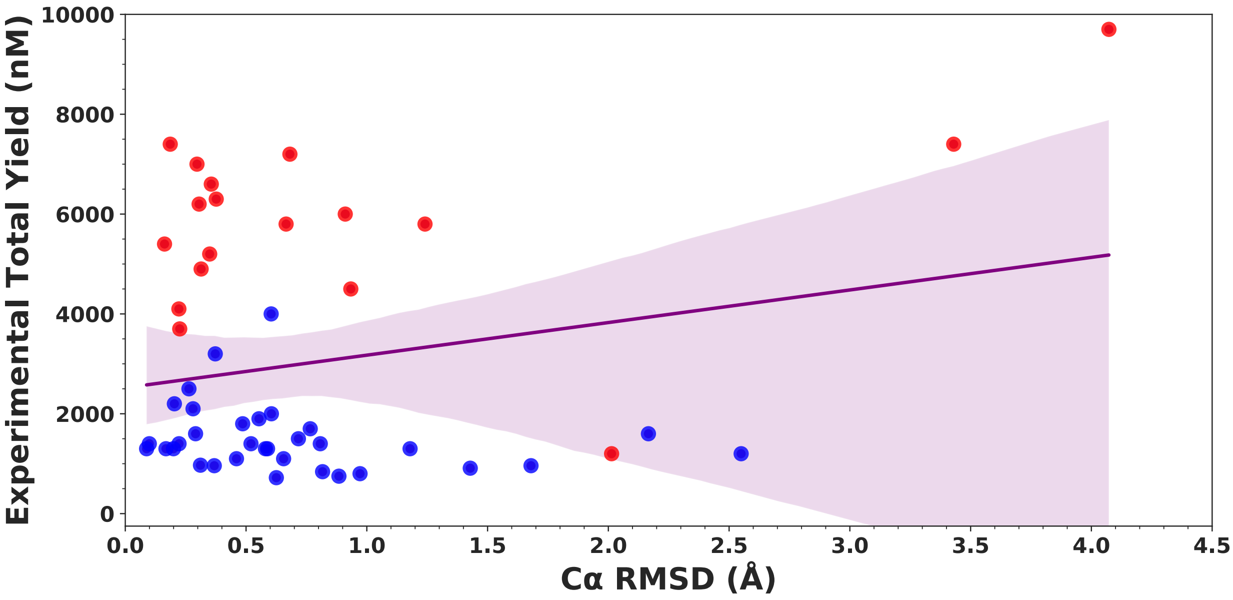


**Supplementary Figure 2.** Plot demonstrating the RMSD distribution between Acd and WT simulations vs experimental total yield data. Points for RecA data are colored red and points for LexA data are colored blue.

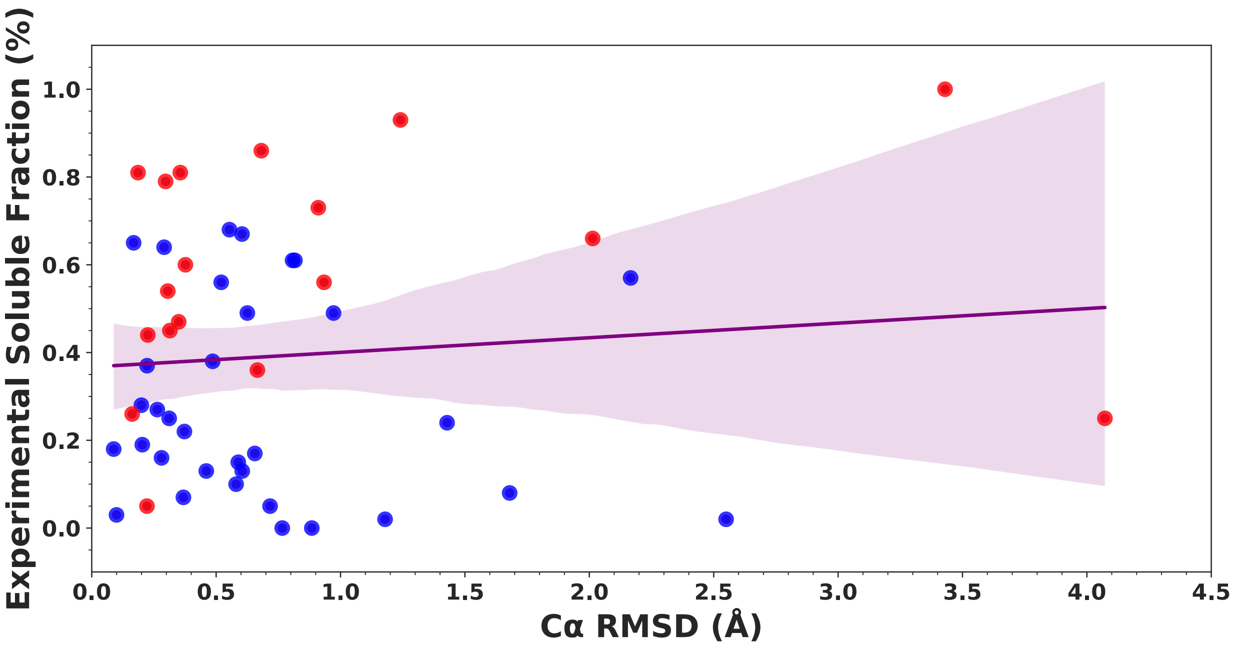


**Supplementary Figure 3.** Plot demonstrating the RMSD distribution between Acd and WT simulations vs experimental soluble fraction data. Points for RecA data are colored red and points for LexA data are colored blue.

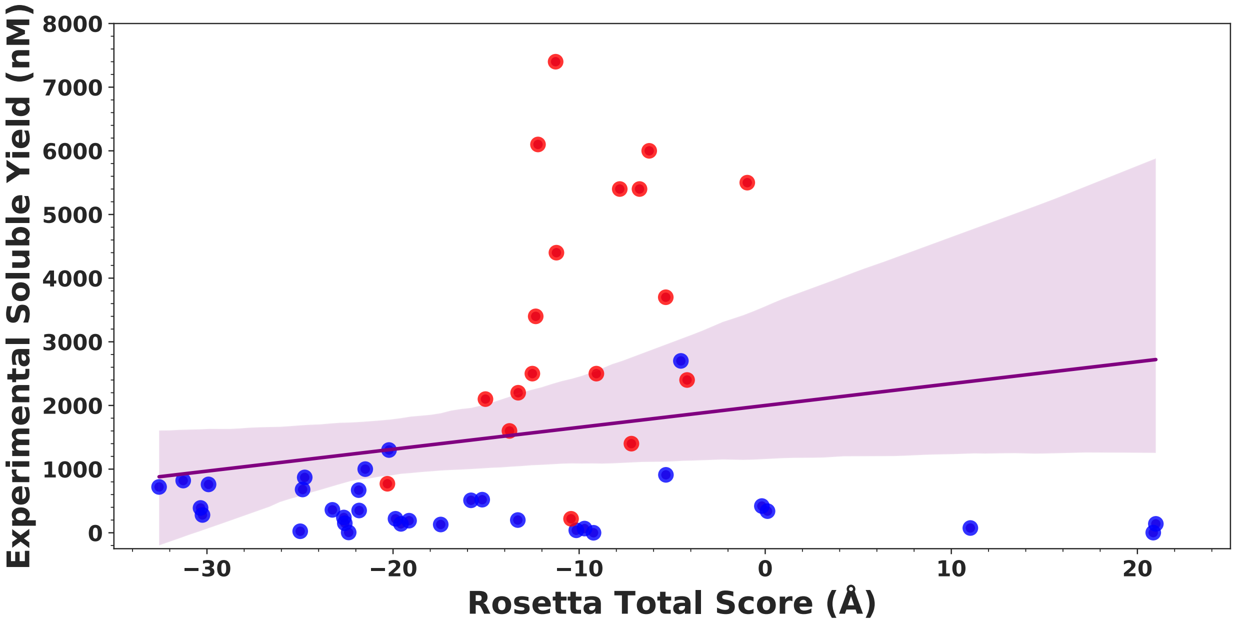


**Supplementary Figure 4.** Plot demonstrating the REU distribution between Acd and WT simulations vs experimental soluble yield data. Points for RecA data are colored red and points for LexA data are colored blue.

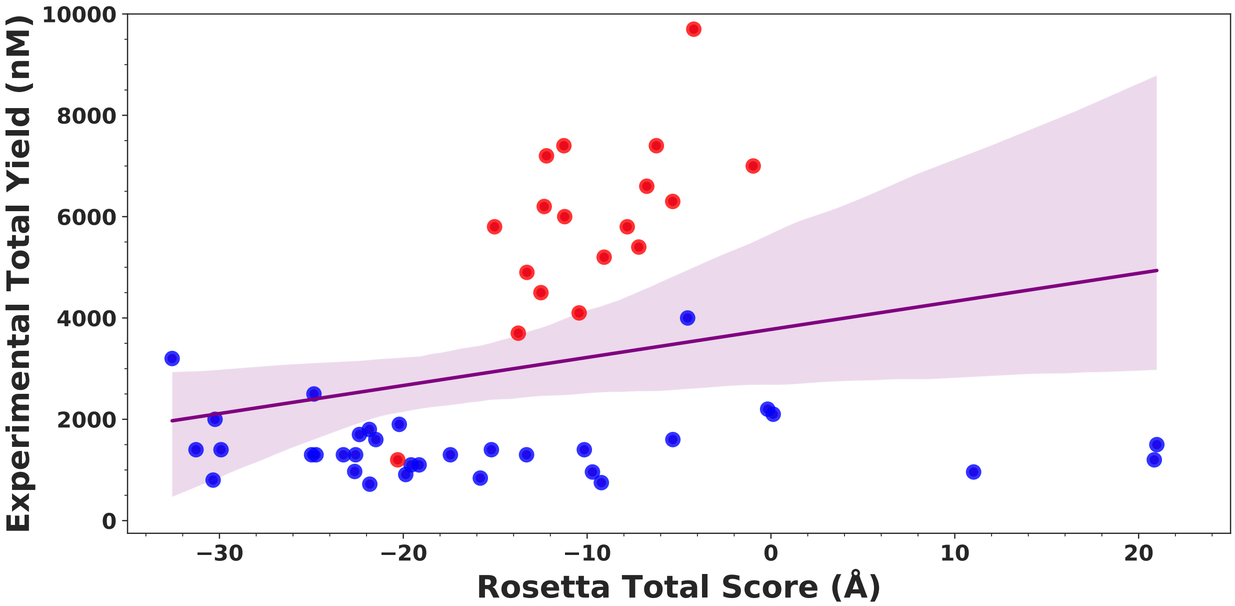


**Supplementary Figure 5.** Plot demonstrating the REU distribution between Acd and WT simulations vs experimental total yield data. Points for RecA data are colored red and points for LexA data are colored blue.

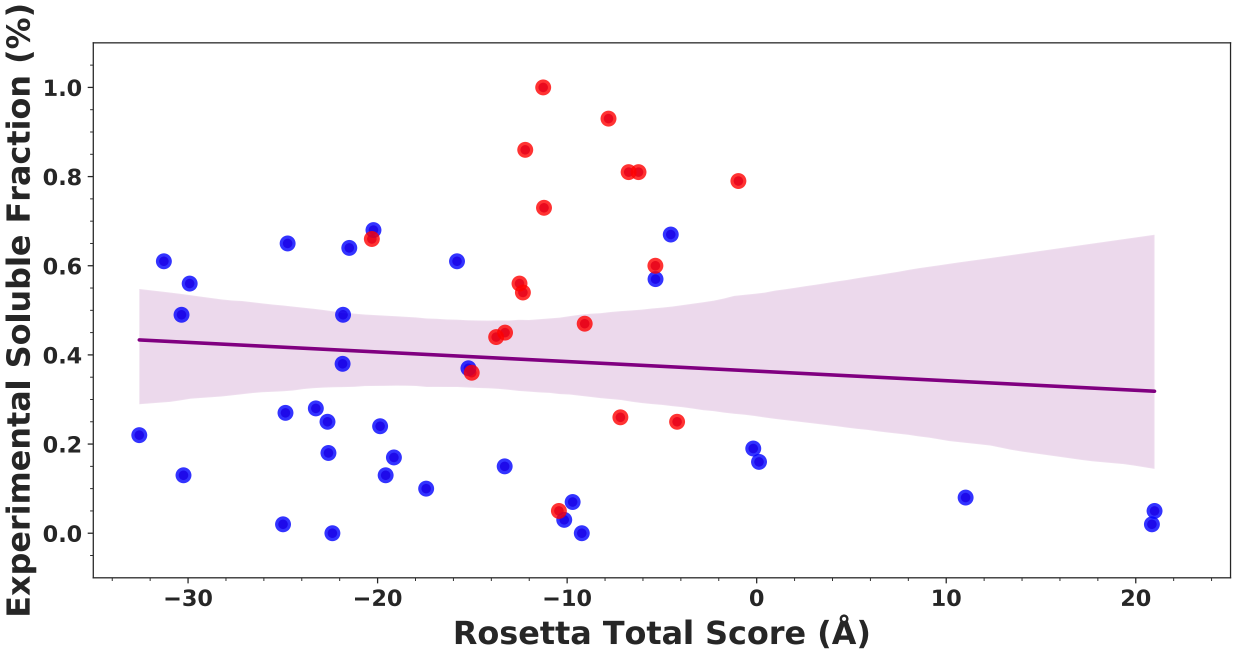


**Supplementary Figure 6.** Plot demonstrating the REU distribution between Acd and WT simulations vs experimental soluble fraction data. Points for RecA data are colored red and points for LexA data are colored blue.

## Feature Analysis

The two feature sets used in this study are the score terms from the Rosetta beta_nov16 score function and the terms found in the following table.

**Supplementary Table 3.** ESF feature descriptions

| Feature | Description |
|---|---|
| np_bb_sc_intra | Mutation site C, $C_\alpha$, and $C_\beta$ contacts with mutation site sidechain carbons within 4 Å |
| p_bb_sc_intra | Mutation site N, O, and NH contacts with mutation sidechain heteroatoms and polar hydrogens within 4 Å |
| np_bb_bb_inter | Mutation site C, $C_\alpha$, and $C_\beta$ contacts with other residue C, $C_\alpha$, and $C_\beta$ atoms within 4 Å |
| p_bb_bb_inter | Mutation site N, O, and NH contacts with other residue N, O, and NH atoms within 4 Å |
| np_bb_sc_inter | Mutation site C, $C_\alpha$, and $C_\beta$ contacts with other residue sidechain carbons within 4 Å |
| p_bb_sc_inter | Mutation site N, O, and NH contacts with other residue sidechain heteroatoms and polar hydrogens within 4 Å |
| np_sc_sc_inter | Mutation site sidechain carbon contacts with other residue sidechain carbons within 4 Å |
| p_sc_sc_inter | Mutation site sidechain heteroatoms and polar hydrogen contacts with other residue sidechain heteroatoms and polar hydrogens within 4 Å |
| np_total | Change in total nonpolar contacts upon mutation |
| p_total | Change in total polar contacts upon mutation |
| total_contacts | Change in total contacts upon mutation |
| kd_vapor_to_water | Sidechain distribution coefficient values for sidechain transfer from vapor to water[7] |
| kd_cyclohexane_to_water | Sidechain distribution coefficient values for sidechain transfer from cyclohexane to water[7] |
| tendency_transmemb_helix | Tendency of amino acid residue to be found in a transmembrane helix[7] |
| tendency_buried | Tendency of amino acid residue to be buried in the interior of a globular protein[7] |
| kd_wet_octanol_to_water | Sidechain distribution coefficient values for side-chain transfer from wet octanol to water[7] |
| kd_water_to_wet_octanol | Sidechain distribution coefficient values for side-chain transfer water to wet octanol[7] |

| | |
|---|---|
| kd_water_to_wet_octanol_adj | Theoretical pentapeptide distribution coefficient values for transfer from water to wet octanol, after adjustment for the effects of occlusion by neighboring residues[7] |
| bilayer_dG2_pH8 | Water to-bilayer partition coefficients for WLXLL peptide where X is the variable amino acid[8] |
| bilayer_dG3_pH8 | Water to-bilayer partition coefficients for X, where X is a variable amino acid[8] |
| helical_propensity | Helical propensity of amino acids normalized to glycine analogue[9] |
| retention_time | Raw peptide retention time on HPLC[9] |
| delta_t_b_R | Change in HPLC retention time of different peptides vs. the glycine analogue[9] |
| t_c_m | The temperature at which half of the a-helix is unfolded[9] |
| delta_t_d_m | Change in melting temperature values between each peptide analogue and that of the glycine analogue[9] |
| blosum62_trp | Score of mutation from an amino acid to tryptophan[10] |
| blosum62_tyr | Score of mutation from an amino acid to tyrosine[10] |
| blosum62_phe | Score of mutation from an amino acid to phenylalanine[10] |
| blosum62_his | Score of mutation from an amino acid to histidine[10] |
| ASA | Accessible surface area from DSSP[11] |
| RSA | Relative accessible surface area from DSSP[11] |

Locally relaxed complexes following mutation to Acd were scored with the beta_nov16 score function where PyRosetta was initialized with the pairwise energy term distance changed from 6 Å to 9 Å. Additionally, the score function was passed to the decompose bb_hb_into_pair_energies energy option and values were recorded on a per residue basis. Scores corresponding to the five local relax trials were averaged for both the Acd and WT structures. Score differences (deltas) were computed by subtracting the averaged Acd scores by the averaged WT scores. Features which

contain the "_Site" suffix are derived from the residue where Acd was incorporated. Features with the "_8A" suffix are derived from the average of all other residues that were designated to move based on the MoveMap in the local relax. Supplementary Table 3 shows the minimum, maximum, mean, and standard deviation of the score deltas used for ML.

**Supplementary Table 4.** RCSF feature vector statistics for Acd simulation score deltas

| Feature | Minimum | Maximum | Mean | SD[a] |
|---|---|---|---|---|
| fa atr_Site | -7.27726 | 1.079198 | -1.594958863 | 2.411328417 |
| fa rep_Site | -0.619908 | 1.87322 | 0.345060471 | 0.568466153 |
| fa sol_Site | -3.55888 | 3.427204 | -0.092241843 | 1.194203379 |
| lk ball_Site | -1.86528 | 2.13022 | 0.072024353 | 0.665871949 |
| lk ball iso_Site | -1.69724 | 6.14483 | 1.018199686 | 1.879585992 |
| lk ball bridge_Site | -0.09687 | 0.09745 | -0.010987137 | 0.032887287 |
| lk ball bridge uncpl_Site | -0.72541 | 0.82046 | -0.074776588 | 0.244973619 |
| fa elec_Site | -1.71069 | 3.31535 | -0.404030902 | 0.721643444 |
| fa intra elec_Site | -0.78131 | 2.39097 | 0.070170157 | 0.425143776 |
| hbond sr bb_Site | -0.141136 | 0.64093 | 0.021098745 | 0.131302268 |
| hbond lr bb_Site | -0.11833 | 0.268592 | 0.003814275 | 0.059146735 |
| omega_Site | -0.603762 | 0.33135 | -0.05169749 | 0.16543746 |
| fa dun dev_Site | -0.60421 | 1.76996 | 0.052755255 | 0.326032301 |
| fa dun rot_Site | -3.18234 | 3.06736 | -0.052563922 | 1.241846764 |
| fa dun semi_Site | -5.12915 | 0.20001 | -0.607660471 | 1.401281154 |
| p aa pp_Site | -0.38255 | 0.80097 | 0.101089686 | 0.23398861 |
| hxl tors_Site | -2.38171 | 0.003534 | -0.093773137 | 0.466086333 |
| rama prepro_Site | -3.389734 | 1.01728 | -0.209784078 | 0.976384669 |
| fa intra atr xover_Site | -1.65992 | 0.18774 | -0.402500941 | 0.590787232 |
| fa intra rep xover_Site | -0.88846 | 0.755428 | 0.010964706 | 0.299195731 |
| fa intra sol xover_Site | -0.93898 | 0.59709 | -0.007120824 | 0.253234145 |
| hbond bb sc_Site | -0.076512 | 1.19086 | 0.048826863 | 0.197999225 |
| hbond sc_Site | -0.34507 | 1.51581 | 0.061363529 | 0.270230674 |

| | | | | |
|---|---|---|---|---|
| residue total score_Site | -12.58673 | 2.158916 | -2.564887804 | 3.120609211 |
| fa atr_8A | -1.750632 | 0.48489 | -0.470670157 | 0.444983063 |
| fa rep_8A | -0.22969 | 0.929078 | 0.123546745 | 0.204277493 |
| fa sol_8A | -0.69745 | 0.657378 | -0.02221702 | 0.30089417 |
| lk ball_8A | -0.29152 | 0.654886 | 0.025330078 | 0.163946665 |
| lk ball iso_8A | -0.75253 | 1.442748 | 0.305896824 | 0.392047644 |
| lk ball bridge_8A | -0.03388 | 0.020528 | -0.00202251 | 0.011150192 |
| lk ball bridge uncpl_8A | -0.3184 | 0.14404 | -0.014054353 | 0.080031492 |
| fa elec_8A | -0.4793 | 0.39896 | -0.101589059 | 0.201487842 |
| fa intra elec_8A | -0.3411 | 0.219504 | 0.018335686 | 0.078125414 |
| pro close_8A | -0.133464 | 0.53565 | 0.013672745 | 0.108126589 |
| hbond_sr_bb_8A | -0.08365 | 0.11867 | 0.000351216 | 0.032321461 |
| hbond lr bb_8A | -0.064964 | 0.138174 | 0.008179412 | 0.033642672 |
| omega_8A | -0.07308 | 0.10032 | 0.009412745 | 0.042115482 |
| fa dun dev_8A | -0.12534 | 0.15336 | 0.021667216 | 0.065511411 |
| fa dun rot_8A | -0.41612 | 0.484196 | 0.025983059 | 0.172567233 |
| fa dun semi_8A | -0.7858 | 0.1922 | -0.145276392 | 0.240073157 |
| p aa pp_8A | -0.1006 | 0.32075 | 0.039800824 | 0.074155419 |
| hxl tors_8A | -0.61228 | 0.24599 | -0.012871725 | 0.096504353 |
| rama prepro_8A | -0.3394 | 0.3624 | -0.003774039 | 0.142596848 |
| fa intra atr xover_8A | -0.366252 | 0.02914 | -0.093786549 | 0.09229524 |
| fa intra rep xover_8A | -0.070422 | 0.12832 | 0.028855137 | 0.042685156 |
| fa intra sol xover_8A | -0.078714 | 0.15604 | 0.009386392 | 0.047326508 |
| hbond bb sc_8A | -0.266424 | 0.19066 | 0.008881333 | 0.075145993 |
| hbond sc_8A | -0.176798 | 0.23728 | 0.015589412 | 0.07371393 |
| ref_8A | -0.26277 | 0.34185 | 0.024140392 | 0.129455713 |
| residue total score_8A | -1.861738 | 0.40139 | -0.654699373 | 0.520996729 |
| total_score | -32.57338 | 20.990798 | -13.5609202 | 11.59259496 |

[a]Standard deviation

The WT and Acd mutant PyRosetta structures were analyzed for changes in relevant contacts as a function of mutation. In addition to these contact-based features, other bioinformatics features such as amino acid measures of hydrophobicity, solvent accessible surface areas, scores from blosum matrices, and conservation were computed. Supplementary Table 9 provides a description of these features and Supplementary Table 10 shows the feature vector statistics (minimum, maximum, mean, and standard deviation). Collectively, we refer to these terms as Empirical Score Functions, or ESFs.

**Supplementary Table 5.** ESF feature vector statistics

| Feature | Minimum | Maximum | Mean | SD[a] |
|---|---|---|---|---|
| np_bb_sc_intra | 0.000 | 4.000 | 1.078 | 1.508 |
| p_bb_sc_intra | -1.000 | 0.000 | -0.020 | 0.140 |
| np_bb_bb_inter | -2.000 | 1.000 | 0.020 | 0.424 |
| p_bb_bb_inter | -4.000 | 8.000 | 0.333 | 1.657 |
| np_bb_sc_inter | -2.000 | 3.000 | 0.039 | 0.799 |
| p_bb_sc_inter | -2.000 | 4.000 | -0.059 | 0.785 |
| np_sc_sc_inter | -2.000 | 11.000 | 1.569 | 2.773 |
| p_sc_sc_inter | -4.000 | 1.000 | -0.196 | 0.775 |
| np_total | -3.000 | 13.000 | 2.706 | 3.961 |
| p_total | -9.000 | 9.000 | 0.059 | 2.158 |
| total_contacts | -6.000 | 14.000 | 2.765 | 4.288 |
| kd_vapor_to_water | -19.920 | 2.390 | -6.581 | 6.955 |
| kd_cyclohexane_to_water | -14.920 | 4.920 | -3.615 | 5.969 |
| tendency_transmemb_helix | -0.600 | 3.490 | 1.405 | 1.319 |
| tendency_buried | -2.71 | 0.24 | -0.964 | 0.851 |
| kd_wet_octanol_to_water | -1.32 | 2.09 | 0.185 | 1.034 |
| kd_water_to_wet_octanol | -2.53 | 2.99 | 0.824 | 1.851 |
| kd_water_to_wet_octanol_adj | -1.71 | 3.64 | 1.086 | 1.68 |
| bilayer_dG2_pH8 | 2.23 | 5.38 | 3.86 | 0.833 |
| bilayer_dG3_pH8 | -2.02 | 1.13 | -0.387 | 0.834 |

| | | | | |
|---|---|---|---|---|
| helical_propensity | -0.83 | 0.96 | 0.405 | 0.411 |
| retention_time | 10.5 | 22.6 | 15.543 | 3.818 |
| delta_t_b_R | -4.3 | 7.8 | 0.743 | 3.818 |
| t_c_m | 41.3 | 73.6 | 57.633 | 8.94 |
| delta_t_d_m | -6 | 26.3 | 10.333 | 8.94 |
| blosum62_trp | -4 | 2 | -2.471 | 1.391 |
| blosum62_tyr | -3 | 7 | -1.412 | 2.08 |
| blosum62_phe | -4 | 6 | -1.882 | 2.215 |
| blosum62_his | -3 | 2 | -1.039 | 1.356 |
| ASA | 0 | 179 | 72.725 | 52.88 |
| RSA | 0 | 0.722 | 0.347 | 0.228 |

[a]Standard deviation

## Descriptive Analysis

In our efforts to identify correlative features which could be used to accurately predict the Acd mutant protein solubility and yield, we observed that our Rosetta feature vectors displayed variance. We sought to quantify whether the identified variances in the feature vectors trend with the experimental parameters. Supplementary Table 4 demonstrates the direct Pearson correlation between each RCSF feature and the three dependent variables.

**Supplementary Table 6.** Correlation of RCSF score deltas with dependent variables from the LexA + RecA set

| Feature | Soluble Yield (R) | Total Yield (R) | Soluble Fraction (R) |
|---|---|---|---|
| fa atr_Site | -0.574 | -0.631 | -0.486 |
| fa rep_Site | 0.382 | 0.449 | 0.336 |
| fa sol_Site | -0.153 | -0.152 | -0.089 |
| lk ball_Site | -0.030 | 0.050 | -0.010 |
| lk ball iso_Site | 0.381 | 0.465 | 0.334 |
| lk ball bridge_Site | -0.527 | -0.587 | -0.246 |
| lk ball bridge uncpl_Site | -0.520 | -0.555 | -0.246 |

| | | | |
|---|---|---|---|
| fa elec_Site | 0.427 | 0.435 | 0.183 |
| fa intra elec_Site | 0.126 | 0.409 | -0.001 |
| hbond sr bb_Site | 0.308 | 0.209 | 0.349 |
| hbond lr bb_Site | -0.179 | -0.187 | -0.248 |
| omega_Site | -0.146 | -0.021 | -0.241 |
| fa dun dev_Site | -0.029 | 0.047 | -0.142 |
| fa dun rot_Site | 0.051 | 0.065 | 0.013 |
| fa dun semi_Site | -0.313 | -0.527 | -0.187 |
| p aa pp_Site | 0.412 | 0.495 | 0.269 |
| hxl tors_Site | -0.238 | -0.288 | -0.132 |
| rama prepro_Site | 0.109 | 0.017 | 0.177 |
| fa intra atr xover_Site | -0.597 | -0.757 | -0.422 |
| fa intra rep xover_Site | -0.050 | 0.090 | -0.061 |
| fa intra sol xover_Site | -0.160 | -0.197 | -0.075 |
| hbond bb sc_Site | 0.464 | 0.507 | 0.255 |
| hbond sc_Site | 0.450 | 0.310 | 0.328 |
| residue total score_Site | -0.474 | -0.533 | -0.434 |
| fa atr_8A | 0.486 | 0.457 | 0.298 |
| fa rep_8A | -0.254 | -0.199 | -0.270 |
| fa sol_8A | -0.347 | -0.349 | -0.205 |
| lk ball_8A | -0.262 | -0.238 | -0.187 |
| lk ball iso_8A | -0.502 | -0.472 | -0.322 |
| lk ball bridge_8A | -0.408 | -0.469 | -0.165 |
| lk ball bridge uncpl_8A | -0.501 | -0.497 | -0.219 |
| fa elec_8A | 0.060 | 0.155 | -0.089 |
| fa intra elec_8A | -0.093 | -0.079 | -0.126 |
| pro close_8A | -0.004 | -0.022 | 0.093 |
| hbond_sr_bb_8A | -0.033 | -0.168 | -0.012 |
| hbond lr bb_8A | -0.171 | -0.193 | -0.180 |
| omega_8A | -0.150 | -0.139 | -0.201 |
| fa dun dev_8A | -0.284 | -0.324 | 0.039 |

| | | | |
|---|---|---|---|
| fa dun rot_8A | -0.237 | -0.251 | -0.109 |
| fa dun semi_8A | 0.391 | 0.468 | 0.212 |
| p aa pp_8A | -0.074 | -0.135 | -0.130 |
| hxl tors_8A | 0.226 | 0.214 | 0.029 |
| rama prepro_8A | 0.403 | 0.272 | 0.500 |
| fa intra atr xover_8A | 0.557 | 0.664 | 0.311 |
| fa intra rep xover_8A | -0.071 | -0.034 | 0.009 |
| fa intra sol xover_8A | -0.127 | -0.198 | 0.024 |
| hbond bb sc_8A | -0.172 | -0.060 | -0.146 |
| hbond sc_8A | 0.224 | 0.159 | 0.118 |
| ref_8A | -0.117 | -0.062 | -0.151 |
| residue total score_8A | 0.453 | 0.493 | 0.210 |
| total_score | 0.205 | 0.268 | -0.089 |

In the same fashion as the Rosetta features, we investigated the correlation of each of the ESF features with the dependent variables. Supplementary Table 11 demonstrates the direct Pearson correlation between each contact feature and the three dependent variables.

**Supplementary Table 7.** Correlation of ESF features with dependent variables from the LexA + RecA set

| Feature | Soluble Yield (R) | Total Yield (R) | Soluble Fraction (R) |
|---|---|---|---|
| np_bb_sc_intra | 0.695 | 0.798 | 0.503 |
| p_bb_sc_intra | -0.071 | -0.130 | -0.040 |
| np_bb_bb_inter | 0.187 | 0.115 | 0.196 |
| p_bb_bb_inter | -0.087 | -0.010 | -0.044 |
| np_bb_sc_inter | -0.085 | -0.094 | 0.013 |
| p_bb_sc_inter | 0.084 | 0.149 | 0.009 |
| np_sc_sc_inter | 0.421 | 0.376 | 0.390 |
| p_sc_sc_inter | -0.423 | -0.328 | -0.322 |
| np_total | 0.562 | 0.560 | 0.488 |
| p_total | -0.193 | -0.080 | -0.149 |

| | | | |
|---|---|---|---|
| total_contacts | 0.423 | 0.477 | 0.376 |
| kd_vapor_to_water | -0.269 | -0.254 | -0.219 |
| kd_cyclohexane_to_water | -0.239 | -0.222 | -0.227 |
| tendency_transmemb_helix | 0.118 | 0.173 | 0.097 |
| tendency_buried | -0.236 | -0.184 | -0.201 |
| kd_wet_octanol_to_water | -0.177 | -0.184 | -0.216 |
| kd_water_to_wet_octanol | 0.043 | -0.107 | 0.042 |
| kd_water_to_wet_octanol_adj | 0.055 | 0.155 | 0.077 |
| bilayer_dG2_pH8 | -0.069 | -0.207 | -0.020 |
| bilayer_dG3_pH8 | -0.070 | -0.207 | -0.022 |
| helical_propensity | 0.074 | 0.014 | -0.004 |
| retention_time | -0.085 | -0.151 | -0.093 |
| delta_t_b_R | -0.085 | -0.151 | -0.093 |
| t_c_m | 0.082 | -0.026 | -0.016 |
| delta_t_d_m | 0.082 | -0.026 | -0.016 |
| blosum62_trp | 0.023 | -0.054 | 0.105 |
| blosum62_tyr | 0.008 | -0.017 | 0.030 |
| blosum62_phe | -0.088 | -0.130 | -0.066 |
| blosum62_his | 0.168 | 0.190 | 0.167 |
| ASA | 0.242 | 0.223 | 0.241 |
| RSA | 0.163 | 0.151 | 0.224 |

**Dimensionality Reduction**

Descriptive analysis of the experimental data using RCSF features proved to be effective. However, we were interested in the predictive capability of these features and, due to the size of the experimental dataset, the total number of features must be reduced to avoid overfitting the dataset. Features for machine learning were selected using the SelectKBest module from sklearn.feature_selection utilizing the mutual_info_classif score function in univariate analysis. Given the size of the dataset we set an upper limit of 10 features to reduce any overfitting. Supplementary Tables 5 demonstrates the number and identity of the features that were selected for each model based on the highest accuracy in forward feature selection.

**Supplementary Table 8.** Selected features for soluble yield RCSFs

| Feature | Soluble Yield BNB | Total Yield KRR | Soluble Fraction NuSVC |
|---|---|---|---|
| Scaler/Transformer Used | **Standard** | **Power** | **Power** |
| fa atr _Site | **Selected** | **Selected** | **Selected** |
| fa rep _Site | N/A | N/A | N/A |
| fa sol _Site | N/A | N/A | N/A |
| lk ball _Site | N/A | N/A | N/A |
| lk ball iso _Site | N/A | N/A | N/A |
| lk ball bridge _Site | **Selected** | N/A | N/A |
| lk ball bridge uncpl _Site | N/A | N/A | **Selected** |
| fa elec _Site | N/A | N/A | N/A |
| fa intra elec _Site | N/A | N/A | **Selected** |
| hbond sr bb _Site | N/A | N/A | N/A |
| hbond lr bb _Site | **Selected** | N/A | N/A |
| omega _Site | N/A | N/A | **Selected** |
| fa dun dev _Site | N/A | N/A | N/A |
| fa dun rot _Site | N/A | N/A | **Selected** |
| fa dun semi _Site | N/A | N/A | N/A |
| p aa pp _Site | **Selected** | N/A | N/A |
| hxl tors _Site | N/A | N/A | N/A |
| rama prepro _Site | N/A | N/A | N/A |
| fa intra atr xover _Site | N/A | **Selected** | N/A |
| fa intra rep xover _Site | N/A | N/A | N/A |
| fa intra sol xover _Site | **Selected** | N/A | **Selected** |
| hbond bb sc _Site | N/A | N/A | N/A |
| hbond sc _Site | N/A | N/A | N/A |
| residue total score _Site | **Selected** | **Selected** | N/A |
| fa atr _8A | N/A | N/A | N/A |
| fa rep _8A | N/A | N/A | N/A |
| fa sol _8A | N/A | N/A | N/A |
| lk ball _8A | N/A | N/A | **Selected** |
| lk ball iso _8A | N/A | N/A | N/A |
| lk ball bridge _8A | N/A | N/A | N/A |
| lk ball bridge uncpl _8A | N/A | N/A | N/A |
| fa elec _8A | N/A | N/A | N/A |

| | | | |
|---|---|---|---|
| fa intra elec _8A | N/A | N/A | N/A |
| pro close _8A | N/A | N/A | N/A |
| hbond_sr_bb _8A | N/A | N/A | N/A |
| hbond lr bb _8A | N/A | N/A | N/A |
| omega _8A | **Selected** | N/A | N/A |
| fa dun dev _8A | N/A | N/A | N/A |
| fa dun rot _8A | N/A | N/A | **Selected** |
| fa dun semi _8A | N/A | N/A | N/A |
| p aa pp _8A | N/A | N/A | N/A |
| hxl tors _8A | N/A | N/A | N/A |
| rama prepro _8A | N/A | N/A | N/A |
| fa intra atr xover _8A | **Selected** | **Selected** | **Selected** |
| fa intra rep xover _8A | N/A | N/A | N/A |
| fa intra sol xover _8A | N/A | N/A | N/A |
| hbond bb sc _8A | N/A | N/A | N/A |
| hbond sc _8A | N/A | N/A | N/A |
| ref _8A | N/A | **Selected** | N/A |
| residue total score _8A | N/A | N/A | N/A |
| total score | N/A | N/A | N/A |

In an effort to reduce model overfitting, the ESF features were put into feature selection using the SelectKBest module, mutual_info_classif score function, and limited to 10 features. Accuracies from untuned model cross validation from forward feature selection were used to select the final set of features displayed Supplementary Table 12.

**Supplementary Table 9.** ESF selected features for soluble yield models

| Feature | Soluble Yield SVC POL3 | Total Yield QDA | Soluble Fraction KNN |
|---|---|---|---|
| Scaler/Transformer Used | **MinMax** | **Quantile** | **Power** |
| np_bb_sc_intra | **Selected** | **Selected** | N/A |
| p_bb_sc_intra | N/A | **Selected** | N/A |
| np_bb_bb_inter | N/A | **Selected** | N/A |
| p_bb_bb_inter | N/A | N/A | N/A |
| np_bb_sc_inter | **Selected** | **Selected** | **Selected** |
| p_bb_sc_inter | N/A | N/A | N/A |
| np_sc_sc_inter | **Selected** | N/A | **Selected** |
| p_sc_sc_inter | N/A | N/A | N/A |
| np_total | **Selected** | **Selected** | **Selected** |
| p_total | N/A | N/A | N/A |
| total_contacts | **Selected** | **Selected** | **Selected** |
| kd_vapor_to_water | N/A | N/A | N/A |
| kd_cyclohexane_to_water | N/A | N/A | N/A |
| tendency_transmemb_helix | N/A | N/A | N/A |
| tendency_buried | N/A | N/A | N/A |
| kd_wet_octanol_to_water | N/A | N/A | N/A |
| kd_water_to_wet_octanol | N/A | N/A | N/A |
| kd_water_to_wet_octanol_adj | N/A | **Selected** | N/A |
| bilayer_dG2_pH8 | N/A | N/A | N/A |
| bilayer_dG3_pH8 | N/A | N/A | N/A |
| helical_propensity | N/A | N/A | N/A |
| retention_time | N/A | N/A | N/A |
| delta_t_b_R | N/A | N/A | N/A |
| t_c_m | N/A | N/A | N/A |
| delta_t_d_m | N/A | N/A | **Selected** |
| blosum62_trp | N/A | N/A | N/A |
| blosum62_tyr | N/A | N/A | N/A |
| blosum62_phe | N/A | N/A | N/A |
| blosum62_his | **Selected** | N/A | **Selected** |
| ASA | **Selected** | **Selected** | N/A |
| RSA | **Selected** | **Selected** | **Selected** |

## Tuning Parameters

Although we limited the number of features for machine learning to avoid overfitting, we sought to demonstrate both the efficacy and practicality of our models through accurate prediction of a holdout dataset which faithfully represented both LexA and RecA, secondary structure, and tolerated and untolerated positions. Prediction of never-before-seen data in the holdout set eliminates the concern of overfitting due to hyperparameter optimization alone. Supplementary Table 6 show the final values of the parameters of each model determined by exhaustive grid search.

**Supplementary Table 10.** RCSFs tuning parameters for all three dependent variables

| Model | Nu | kernel | gamma | class_weight | tol | alpha |
|---|---|---|---|---|---|---|
| **Soluble Yield BNB** | N/A | N/A | N/A | N/A | N/A | 1 |
| **Total Yield KRR** | N/A | N/A | N/A | N/A | N/A | 1 |
| **Soluble Frac NuSVC** | 0.5 | rbf | auto | balanced | 0.001 | N/A |

Column headings: Nu (regularization parameter), kernel (kernel type), gamma (kernel coefficient), class_weight (parameter which dictates magnitude of loss based on class distribution), tol (tolerance of optimization), and alpha(in BNB alpha is the smoothing parameter, in KRR it is the regularization parameter).

All models following feature selection were tuned using exhaustive grid searching in sklearn. Grid searching was performed with stratified 5-fold cross validation. Supplementary Table 13 displays the final parameter values from the tuned ESF models.

**Supplementary Table 11.** ESF tuning parameters for all three dependent variables

| Model | kernel | degree | gamma | C | tol | reg_param | n_neighbors | p | weights |
|---|---|---|---|---|---|---|---|---|---|
| **Soluble Yield SVC POL3** | poly | 3 | scale | 0.8 | 0.001 | N/A | N/A | N/A | N/A |
| **Total Yield QDA** | N/A | N/A | N/A | N/A | 0.0001 | 0.0 | N/A | N/A | N/A |
| **Soluble Frac KNN** | N/A | N/A | N/A | N/A | N/A | N/A | 5 | 2 | uniform |

Column headings: kernel (model kernel), degree (the polynomial power of the kernel), gamma (kernel coefficient), C (inverse regularization parameter), tol (tolerance of optimization), reg_param (QDA regularization parameter), n_neighbors (number of neighbors for KNN), p (power parameter for KNN), and weights (weight function used for KNN).

## Feature Importance

In addition to investigating the feature importance of the soluble fraction models, we also computed feature importance values for the soluble yield and total yield models. Interestingly, the soluble yield and soluble fraction models are very similar in number and type of features, while the total yield model is five features, heavily biased towards Lennard Jones attraction. It is not surprising that feature importance is different in the total yield model as it is a completely different experimental observable. Supplementary Tables 7 and 8 display the normalized feature importance values for the soluble and total yield RCSFs.

**Supplementary Table 12.** Normalized feature importance for soluble yield BNB RCSF

| RCSF Feature | Normalized Feature Importance (%) |
|---|---|
| hbond_lr_bb_Site | 100.00 |
| omega_8A | 37.05 |
| fa_atr_Site | 28.92 |
| residue_total_score_Site | 28.92 |
| p_aa_pp_Site | 25.51 |
| fa_intra_sol_xover_Site | 17.02 |
| lk_ball_bridge_Site | 17.02 |
| fa_intra_atr_xover_8A | 12.39 |

**Supplementary Table 13.** Normalized feature importance for total yield KRR RCSF

| RCSF Feature | Normalized Feature Importance (%) |
|---|---|
| fa_intra_atr_xover _8A | 100.00 |
| fa_atr _Site | 93.52 |
| ref_8A | 61.87 |
| residue_total_score_Site | 20.15 |
| fa_intra_atr_xover _Site | 13.69 |

In addition to investigating the feature importance of the soluble fraction ESF model, we also computed feature importance values for the soluble yield and total yield ESF models. Interestingly, the soluble yield and soluble fraction models are very similar in number and type of features, while the total yield model has only two features, albeit ones that are shown to be important in the other analyses. It is not surprising that feature importance is different in the total yield model as it is a completely different experimental observable.

**Supplementary Table 14.** Normalized feature importance for soluble yield SVC POL3 ESF

| ESF Feature | Normalized Feature Importance (%) |
|---|---|
| np_total | 100.00 |
| np_bb_sc_intra | 67.22 |
| kd_wet_octanol_to_water | 45.67 |
| np_sc_sc_inter | 36.26 |
| tendency_buried | 26.74 |
| p_sc_sc_inter | 10.77 |
| tendency_transmemb_helix | 5.47 |
| blosum62_phe | 2.32 |
| kd_cyclohexane_to_water | 1.11 |

**Supplementary Table 15.** Normalized feature importance for total yield QDA ESF

| ESF Feature | Normalized Feature Importance (%) |
|---|---|
| ASA | 100.00 |
| total_contacts | 88.01 |
| np_bb_sc_intra | 62.31 |
| np_total | 38.01 |
| np_bb_bb_inter | 34.72 |
| np_bb_sc_inter | 9.68 |
| kd_water_to_wet_octanol_adj | 7.93 |
| RSA | 3.79 |
| p_bb_sc_intra | 1.46 |

## References


1 Pedregosa, F. *et al.* Scikit-learn: Machine Learning in Python. *Journal of Machine Learning Research* **12**, 2825-2830 (2011).

2 Hostetler, Z. M. *et al.* Systematic Evaluation of Soluble Protein Expression Using a Fluorescent Unnatural Amino Acid Reveals No Reliable Predictors of Tolerability. *Acs Chemical Biology* **13**, 2855-2861, doi:10.1021/acschembio.8b00696 (2018).

3 Luo, Y. *et al.* Crystal structure of LexA: A conformational switch for regulation of self-cleavage. *Cell* **106**, 585-594, doi:10.1016/s0092-8674(01)00479-2 (2001).

4 Chen, Z. C., Yang, H. J. & Pavletich, N. P. Mechanism of homologous recombination from the RecA-ssDNA/dsDNA structures. *Nature* **453**, 489-U483, doi:10.1038/nature06971 (2008).

5 Renfrew, P. D., Choi, E. J., Bonneau, R. & Kuhlman, B. Incorporation of Noncanonical Amino Acids into Rosetta and Use in Computational Protein-Peptide Interface Design. *PLoS One* **7**, 15, doi:10.1371/journal.pone.0032637 (2012).

6 Dietterich, T. G. Ensemble methods in machine learning. *Multiple Classifier Systems* **1857**, 1-15, doi:10.1007/3-540-45014-9_1 (2000).

7 Wolfenden, R. Experimental measures of amino acid hydrophobicity and the topology of transmembrane and globular proteins. *Journal of General Physiology* **129**, 357-362, doi:10.1085/jgp.200709743 (2007).

8 Wimley, W. C. & White, S. H. Experimentally determined hydrophobicity scale for proteins at membrane interfaces. *Nature Structural Biology* **3**, 842-848, doi:10.1038/nsb1096-842 (1996).

9 Monera, O. D., Sereda, T. J., Zhou, N. E., Kay, C. M. & Hodges, R. S. Relationship of Sidechain Hydrophobicity and alpha-Helical Propensity on the Stability of the Single-stranded Amphipathic alpha-Helix. *Journal of Peptide Science* **1**, 319-329, doi:10.1002/psc.310010507 (1995).

10 Henikoff, S. & Henikoff, J. G. PERFORMANCE EVALUATION OF AMINO-ACID SUBSTITUTION MATRICES. *Proteins-Structure Function and Bioinformatics* **17**, 49-61, doi:10.1002/prot.340170108 (1993).

11 Colloch, N., Etchebest, C., Thoreau, E., Henrissat, B. & Mornon, J. P. COMPARISON OF 3 ALGORITHMS FOR THE ASSIGNMENT OF SECONDARY STRUCTURE IN PROTEINS - THE ADVANTAGES OF A CONSENSUS ASSIGNMENT. *Protein Engineering* **6**, 377-382, doi:10.1093/protein/6.4.377 (1993).
